# Use of an emulated trial to investigate the association between use of nitrogen-based bisphosphonates and risk of epithelial ovarian cancer

**DOI:** 10.1093/ije/dyae108

**Published:** 2024-08-12

**Authors:** Karen M Tuesley, Katrina Spilsbury, Penelope M Webb, Sallie-Anne Pearson, Peter Donovan, Michael D Coory, Christopher B Steer, Louise M Stewart, Nirmala Pandeya, Melinda M Protani, Suzanne Dixon-Suen, Louise Marquart-Wilson, Susan J Jordan

**Affiliations:** School of Public Health, Faculty of Medicine, University of Queensland, Brisbane, QLD, Australia; Population Health Program, QIMR Berghofer Medical Research Institute, Brisbane, QLD, Australia; Institute for Health Research, University of Notre Dame Australia, Fremantle, WA, Australia; School of Public Health, Faculty of Medicine, University of Queensland, Brisbane, QLD, Australia; Population Health Program, QIMR Berghofer Medical Research Institute, Brisbane, QLD, Australia; School of Population Health, University of New South Wales, Sydney, NSW, Australia; Centre of Research Excellence in Medicines Intelligence, University of New South Wales, Sydney, NSW, Australia; Clinical Pharmacology Department, Royal Brisbane and Women’s Hospital, Brisbane, QLD, Australia; Faculty of Medicine, University of Queensland, Brisbane, QLD, Australia; Faculty of Medicine, Dentistry and Health Sciences, University of Melbourne, Melbourne, VIC, Australia; Border Medical Oncology, Albury-Wodonga Regional Cancer Centre, Albury, NSW, Australia; University of NSW Rural Clinical School, Albury Campus, Albury, NSW, Australia; School of Population and Global Health, University of Western Australia, Perth, WA, Australia; School of Public Health, Faculty of Medicine, University of Queensland, Brisbane, QLD, Australia; Population Health Program, QIMR Berghofer Medical Research Institute, Brisbane, QLD, Australia; School of Public Health, Faculty of Medicine, University of Queensland, Brisbane, QLD, Australia; Institute for Physical Activity and Nutrition, Deakin University, Geelong, VIC, Australia; Cancer Epidemiology Division, Cancer Council Victoria, Melbourne, VIC, Australia; School of Public Health, Faculty of Medicine, University of Queensland, Brisbane, QLD, Australia; Clinical Malaria Group, QIMR Berghofer Medical Research Institute, Brisbane, QLD, Australia; School of Public Health, Faculty of Medicine, University of Queensland, Brisbane, QLD, Australia; Population Health Program, QIMR Berghofer Medical Research Institute, Brisbane, QLD, Australia

**Keywords:** Epithelial ovarian cancer, serous, nitrogen-based bisphosphonates, bisphosphonates, emulated trial

## Abstract

**Background:**

Epithelial ovarian cancer (EOC) is the eighth most common cancer in women, with poor survival outcomes. Observational evidence suggests that nitrogen-based bisphosphonate (NBB) use may be associated with reduced risk of EOC, particularly the endometrioid and serous histotypes; however, confounding by indication is a concern. An alternative approach to investigate the chemo-preventive potential of NBBs is to emulate a target trial by identifying all women who initiate use of NBBs and investigate the risk of EOC for continued users compared with discontinued users.

**Methods:**

Using population-based linked data, we identified all Australian women aged over 50 years who first used NBBs over 2004–12. We used the year after first use to define treatment for each woman as either continued or discontinued use. We emulated randomization using stabilized inverse probability weights to balance the treatment groups using covariates including age, comorbidities and socioeconomic status. We followed women from treatment assignment until EOC diagnosis, death or 31 December 2013. We assessed the risk of EOC (overall and by histotype) using flexible parametric time-to-event models allowing for time-varying effects, and produced time-varying coefficients.

**Results:**

Of the 313 383 women in the study, 472 were diagnosed with EOC during follow-up (261 serous EOC), with an average age at diagnosis of 72 years. Continued use of NBBs was associated with reduced risk of EOC overall (HR = 0.87, 95% CI: 0.69, 1.10), and serous EOC (HR = 0.71, 95% CI: 0.53, 0.96), compared with discontinued treatment, with estimates remaining constant over the 9-year follow-up.

**Conclusions:**

Results from our emulated trial suggest that in women who initiated NBB treatment, those who continued use had 13% and 29% lower hazards of being diagnosed with EOC overall and serous EOC, respectively, compared with women who discontinued use.

Key Messages

- We used Australian population-based linked data to perform an emulated trial with the novel approach of assessing the impact of continued or discontinued use of nitrogen-based bisphosphonate medication by including only women indicated for use.
- In women aged over 50 years who initiated nitrogen-based bisphosphonates treatment, those who continued use had a 29% lower hazard of being diagnosed with serous EOC, compared with women who discontinued use.
- The reduction in risk appeared to persist over the 9 years of follow-up.

## Introduction

Epithelial ovarian cancer (EOC) is the eighth most commonly diagnosed cancer in women worldwide, with poor 5-year survival or less than 50%.^1^ Most established risk factors are not readily modifiable,^2^ so other prevention options are needed, one being the repurposing of existing chronic disease medicines.^3^^,^^4^ There is evidence from observational studies that use of nitrogen-based bisphosphonates (NBBs), a medicine group used to treat osteoporosis, may be associated with a reduced risk of EOC, particularly the endometrioid and serous histotypes.^5^^,^^6^

One of the challenges of investigating chronic disease medicine use and risk of cancer in the observational setting is that there are often differences between those who are and are not prescribed the medicines, which could lead to bias due to confounding by indication.^7^ Randomized controlled trials (RCTs) can achieve balance between exposure groups and eliminate this bias, but conducting RCTs to investigate prevention of uncommon cancers such as EOC is generally not feasible due to the very large sample size and long follow-up required to detect a difference of interest. An alternative is to use large-scale observational administrative data to emulate a trial.^8^ Emulated trials using administrative health records have shown similar results to RCTs where the design and measurement were closely aligned.^9^

Prior studies have used emulated trials to assess the effectiveness of medical products, with improved validity achieved when indication for use is similar between treatment groups.^10^ Women without osteoporosis are unlikely to use NBBs and have a very low (and possibly zero) probability of being assigned to a NBB treatment group in an emulated trial. Statistical methods, such as inverse probability of treatment weighting, are therefore unlikely to completely overcome confounding by indication in comparisons of use and no use of NBBs. An alternative is to identify women who initiate NBB use, therefore all have the indication, then define treatment groups as those who continue with treatment compared with those who discontinue use after a short time. Whereas this approach is answering a slightly different question from previous observational studies,^5^^,^^6^ it may provide insights as to whether the medicines have an association with EOC risk, independent of indication for use.

We conducted an emulated trial using Australian population-based linked data, to investigate EOC risk for women aged 50 years and over who were dispensed NBBs and either continued or discontinued use. We hypothesized that if the relationship between NBBs and EOC is causal, and longer duration of use is beneficial, then we would observe a reduced risk of EOC for women with continued use of NBBs compared with those who discontinued use.

## Methods

### Eligibility criteria

The details of the hypothetical target trial underlying our emulated trial design are described in Supplementary Table S1 (available as Supplementary data at *IJE* online). Our study population was derived from a dataset including all adult women enrolled for Medicare, Australia’s universal health insurance scheme, between July 2002 and December 2013. All Australian citizens and permanent residents are eligible for Medicare, therefore essentially all Australian women are included on the Medicare Enrolments File. Records for all women were linked to the Pharmaceutical Benefits Scheme (PBS), the Australian Cancer Database and the National Death Index. From this, we identified all women aged 50 years and older who were first dispensed an NBB between January 2004 and December 2012, and defined this date as the baseline.

The PBS provides subsidised medicines for most medicines in Australia,^11^ and the PBS data for our study included details of all bisphosphonate medicines dispensed from 1 July 2002 onwards. We used the Anatomical Therapeutic Chemical classification system to identify all women dispensed NBBs (Supplementary Table S2, available as Supplementary data at *IJE* online). To avoid including prevalent users whose duration of bisphosphonate use was unknown, we excluded women who were dispensed any osteoporosis medicine between July 2002 and December 2003.

The Australian Cancer Database provided information about all cancer diagnoses from 1982 through 2013. Women with any cancer diagnosis prior to their first NBB use were excluded from the analysis, as we were interested in NBB prescribed for non-cancer indications. We also excluded women who enrolled for Medicare after 1 July 2002 as adults, because the majority would have been immigrants with incomplete cancer histories.

### Treatment groups

We used PBS dispensing records for the year after baseline to assign treatment groups^8^ (Supplementary Figure S1, available as Supplementary data at *IJE* online). We calculated the total defined daily dose of NBBs during that year for each eligible woman using the World Health Organization Collaborating Centre for Drug Statistics Methodology,^12^ PBS item codes (Supplementary Table S1, available as Supplementary data at *IJE* online) and the quantity of each PBS item dispensed. Women were included in the ‘discontinued use’ group if they had no further NBBs dispensed after 6 months from the initial dispensing date (i.e. during the period 6–12 months after baseline) and were dispensed no more than 168 defined daily doses in the first year (equivalent to 6 months with 28 defined daily doses/month). Women included in the ‘continued use’ group therefore had more than 168 defined daily doses in the first year or an NBB dispensed between 6 and 12 months after the initial dispensing date. Zoledronic acid is usually administered once per year,^13^ therefore all women who had this dispensed were included in the ‘continued use’ group. Women who were diagnosed with any cancer or who died during the year after baseline were excluded.

### Outcome

We followed women from 1 year after first NBB use until EOC diagnosis, death or 31 December 2013, whichever came first. We used the International Classification of Diseases for Oncology topography codes to ascertain ovarian cancer (including fallopian tube and primary peritoneal cancer, Supplementary Table S2, available as Supplementary data at *IJE* online), and morphology codes to identify EOC. We classified EOC histotype using the criteria from the 2016 CONCORD-2 study (Supplementary Table S3, available as Supplementary data at *IJE* online)^14^ but did not have sufficient information to distinguish between low- and high-grade serous carcinoma.

### Other variables

We defined several pre-baseline (prior to first NBB use) characteristics for each woman. We defined age at baseline and birth year using records from the Medicare Enrolments File. We used postcode at Medicare enrolment to define state of residence, area-level socioeconomic status using the Socio-Economic Indexes for Areas (SEIFA) Index of Relative Socio-Economic Disadvantage,^15^ and remoteness category using the Accessibility/Remoteness Index of Australia^16^ (Supplementary Methods, available as Supplementary data at *IJE* online).

In Australia, the amount an individual pays (the co-payment) for a prescription medicine listed on the PBS depends on their beneficiary status, defined as either general or concessional if receiving government income support.^11^ The beneficiary category assigned to each dispensed medicine during the year prior to baseline was used to categorize women into one of the following: (i) general prescriptions only: (ii) both general and concessional; (iii) concessional only; or (iv) missing. We used PBS data to identify comorbidities at baseline using Rx-Risk comorbidity categories^17^^,^^18^ for medicines that were available in our data (Supplementary Table S4, available as Supplementary data at *IJE* online). Some low-cost medicines are not subsidized for general beneficiaries in the PBS, and prior to 2012, these were not included in the PBS records (Supplementary Methods, available as Supplementary data at *IJE* online).^19^ We included menopausal hormone therapy (MHT) and other cancer diagnoses as a time-varying covariates, noting that not all MHT medicines are captured in the PBS and therefore we would underestimate use.

### Statistical analysis

We used an intention-to-treat (ITT) analysis to assess EOC risk (overall and by histotype) for continued NBB use compared with discontinued use, based on treatment assignment at 12 months after initial dispensing date (Supplementary Figure S1, available as Supplementary data at *IJE* online). For histotype-specific analyses, we censored follow-up time at the date of diagnosis for women diagnosed with other EOC histotypes. We first generated survival curves using Kaplan–Meier estimates and used Cox regression to produce hazard ratios (HRs) and 95% confidence intervals (CIs), adjusting for age at baseline, to assess the association in an unweighted model (Model 1). We then emulated randomization, using stabilized inverse probability of treatment weights for each participant, by fitting a logistic model for the conditional probability of continued treatment using the following baseline covariates: age, age^2^, year of birth, PBS patient category, comorbidities, state of residence, SEIFA quintile and remoteness of residence. We qualitatively assessed the covariate balance by plotting the standardized mean differences (SMD) and 95% CIs between the treatment groups before and after applying inverse probability weights, and also quantitatively with differences greater than 10% considered to be an indicator of meaningful imbalance.^20^ Using inverse probability weights to balance the treatment groups, we fitted flexible parametric models allowing for time-varying effects.^21^ We included interactions with time to assess whether the association showed time-dependent effects and produced time-varying coefficients (Model 2). Using this model, we report the HR and 95% CI overall and at 6-month and yearly time points.

We then used inverse probability of time-varying treatment weights (1-year time intervals) in a doubly robust pooled logistic regression model, adjusting for pre-baseline covariates associated with the outcome (risk of EOC): age, MHT use and time-varying covariates: MHT use and another cancer diagnosis (Model 3). We also included baseline comorbidities: ischaemic heart disease: angina, gastro-oesophageal reflux disease (GORD), steroid-responsive diseases and pain. Pooled logistic regression was used to account for time-varying weights.^22^ Analyses were performed in Stata 16.0.^23^

### Sensitivity analyses

We performed several sensitivity analyses to explore potential biases in our main analyses, and the rationale for and results of these are described in detail in the Supplementary Methods (available as Supplementary data at *IJE* online). We performed per-protocol sensitivity analyses to assess the effect of non-adherence to the treatment allocation, by censoring follow-up 12 months after NBB use deviated from the initial treatment allocation (Supplementary Figure S1, available as Supplementary data at *IJE* online). We used fitted flexible parametric proportional hazards models allowing for time-varying effects using inverse probability of treatment weights, and also used inverse probability of censoring weights in a pooled logistic regression model with time-varying covariates (Supplementary Materials, available as Supplementary data at *IJE* online).^24^ Other sensitivity analyses included: (i) excluding users of zoledronic acid in the first 12 months after baseline: (ii) starting follow-up at 2 years after baseline; (iii) restricting to women aged ≤70 years and younger at first NBB use; (iv) modelling death as a competing risk; (v) pancreatic cancer as a negative control cancer outcome; and (vii) excluding continuing users with <168 defined daily doses.

## Results

**Figure 1.**
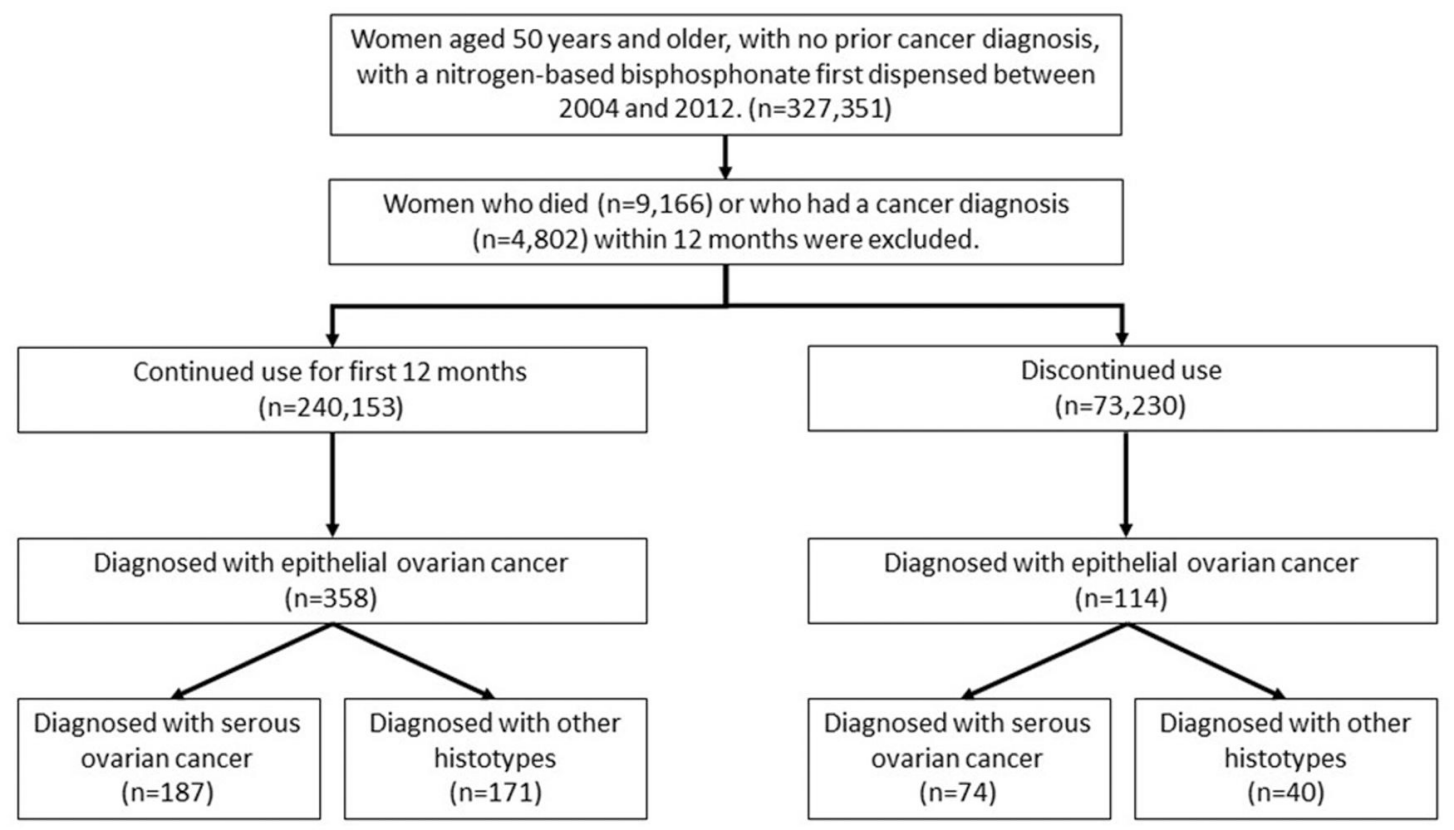
Flow chart of participants in the study

**Figure 2.**
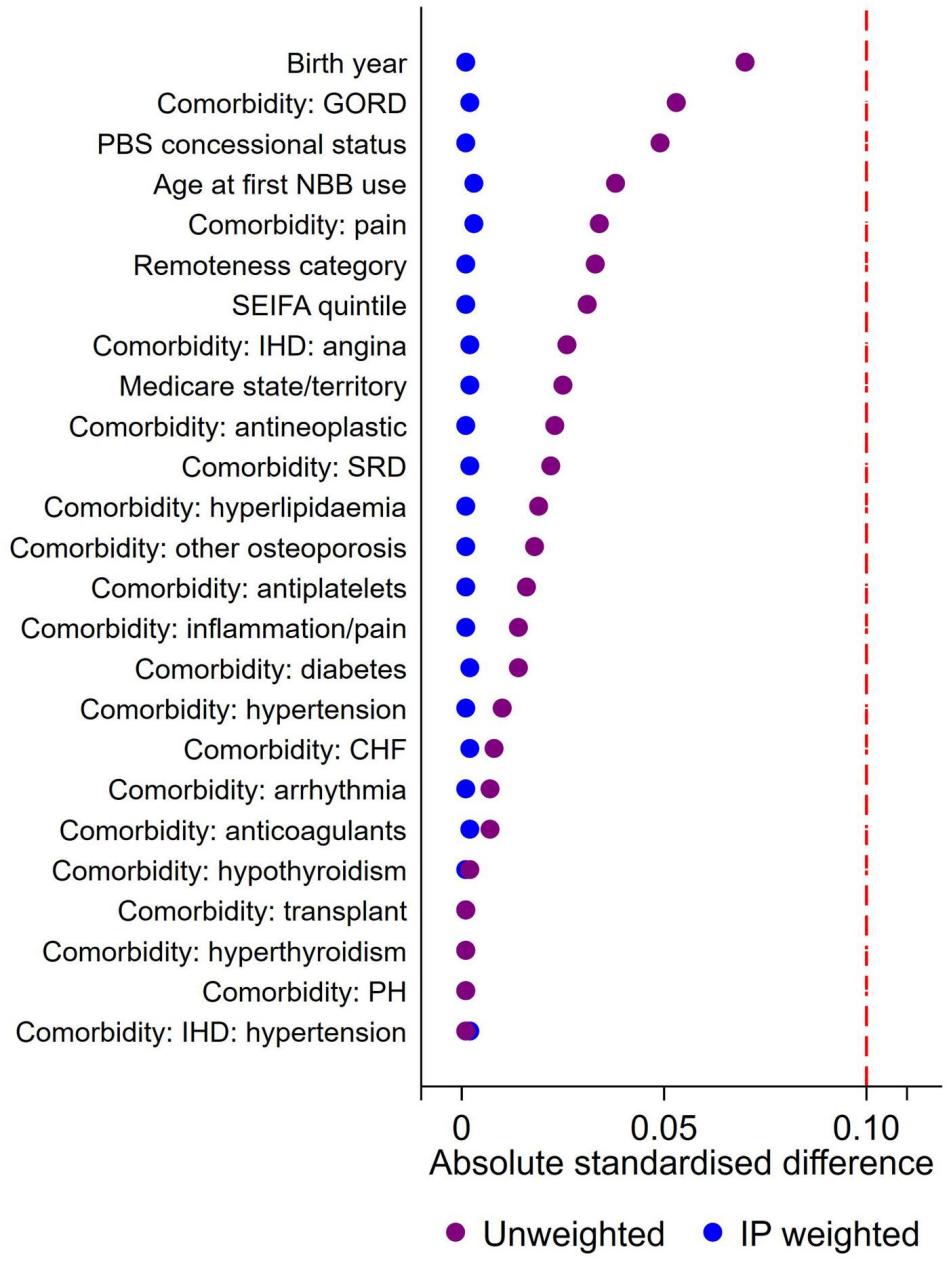
Unmatched and inverse probability of treatment weighted standardized mean differences for each covariate. CHF, congestive heart failure; GORD, gastro-oesophageal reflux disease; IHD, ischaemic heart disease; IPTW, inverse probability of treatment weighted; NBB, nitrogen-based bisphosphonate; PBS, Pharmaceutical Benefits Scheme; PH, pulmonary hypertension; SEIFA, Socio-Economic Indexes for Areas; SRD, steroid-responsive disease

There were 327 351 eligible women aged 50 years or older who were dispensed NBBs for the first time over 2004–12 (Figure 1) with no prior cancer diagnosis. Of these women, 4802 were diagnosed with any cancer and a further 9166 died during the treatment allocation period, and were therefore excluded from the study, leaving 313 383 women. Excluded women were older and with more comorbidities (Table 1; Supplementary Table S6, available as Supplementary data at *IJE* online) compared with included women, (Supplementary Table S7, available as Supplementary data at *IJE* online). Of these, 240 153 met the criteria for the continued use treatment group and 73 230 were in the discontinued use group, with median follow-up of 4.7 [interquartile range (IR): 2.3–6.9] and 4.2 (IR: 2.1–6.3) years, respectively. Table 1 shows the characteristics of the treatment groups, crude SMD^25^ and SMD after applying inverse probability of treatment weights. Figure 2 shows the unmatched and inverse probability weighted SMDs for the variables used in Model 2. All variables had an SMD <10% between treatment groups in the unweighted and inverse probability weighted models; however, Figure 2 shows that weighting improved the balance. Variables with the greatest difference between the treatment groups were birth year (reflecting changing prescribing patterns over time), PBS patient category, age at first NBB use, and Rx-Risk comorbidities: GORD and pain management; 25% of women used MHT during the study.

**Table 1.** Characteristics of treatment groups and standardized mean differences for unmatched and inverse probability of treatment weighted samples

	Unweighted			IP of treatment weighted		
	Discontinued use	Continued use	SMD^a^	Discontinued use	Continued use	SMD^a^
Characteristic	*n *=* *73 230	*n *=* *240 153		*n^b^ *=* *73 191.45	*n^b^ *=* *240 164.75	
Age at first NBB use, mean (SD)	72.2 (11)	72.6 (10)	0.038	72.5 (10)	72.5 (10)	0.003
Birth year, mean (SD)	1936 (11)	1935 (11)	0.070	1935 (11)	1935 (11)	0.001
Medicare registered state/territory (*n* (%))			0.025			0.002
New South Wales	27 517 (38)	92 791 (39)		28 106.6 (38)	92 202.8 (38)	
Australian Capital Territory	1026 (1)	3135 (1)		969.2 (1)	3188.0 (1)	
Victoria	16 924 (23)	54 773 (23)		16 740.1 (23)	54 943.8 (23)	
Queensland	13 515 (19)	44 101 (18)		13 431.8 (18)	44 147.3 (18)	
South Australia/Northern Territory	6774 (9)	21 837 (9)		6674.4 (9)	21 922.7 (9)	
Western Australia	5946 (8)	18 635 (8)		5770.5 (8)	18 847.5 (8)	
Tasmania	1528 (2)	4881 (2)		1498.8 (2)	4912.6 (2)	
SEIFA quintile, *n* (%)			0.031			0.001
1 (most disadvantaged)	16 647 (23)	52 492 (22)		16 131.6 (22)	52 979.0 (22)	
2	15 126 (21)	48 590 (20)		14 893.9 (20)	48 834.6 (20)	
3	14 265 (20)	47 467 (20)		14 396.5 (20)	47 303.8 (20)	
4	13 422 (18)	44 268 (18)		13 463.6 (18)	44 208.3 (18)	
5 (least disadvantaged)	13 402 (18)	46 212 (19)		13 954.7 (19)	45 694.7 (19)	
Missing	368 (1)	1124 (1)		351.1 (1)	1144.4 (1)	
Remoteness category, *n* (%)			0.033			0.001
Major city	51 279 (70)	170 260 (71)		51 720.1 (71)	169 772.2 (71)	
Inner regional	14 760 (20)	48 037 (20)		14 682.4 (20)	48129.6 (20)	
Outer regional	6035 (8)	18 775 (8)		5797.3 (8)	19 015.7 (8)	
Remote/very remote	806 (1)	2010 (1)		658.0 (1)	2157.6 (1)	
Missing	350 (<1)	1071 (<1)		333.7 (1)	1089.6 (1)	
Rx-Risk comorbidities^c^						
PBS concessional status, *n* (%)			0.049			0.001
General status only	8649 (12)	26 349 (11)		8169.2 (11)	26 817.9 (11)	
General and concessional	1950 (3)	6472 (3)		1987.1 (3)	6495.7 (3)	
Concessional only	56 682 (77)	190 226 (79)		57 660.2 (79)	189 221.4 (79)	
Missing	5949 (8)	17 056 (7)		5374.9 (7)	17 629.9 (7)	

IP, inverse probability; NBB, nitrogen-based bisphosphonate; PBS, Pharmaceutical Benefits Scheme; SEIFA, Socio-Economic Indexes for Areas; SD, standard deviation; SMD, standardized mean difference.

a A difference of <0.1 is generally considered acceptable.

b Inverse probability weighted frequencies presented for *n*.

c Details of each comorbidity category are included in Supplementary Table S5, available as Supplementary data at *IJE* online.

There were 472 women diagnosed with EOC during follow-up (Figure 1). The numbers of women diagnosed with endometrioid, clear cell and mucinous histotypes were very small, and therefore analyses were restricted to EOC overall and serous histotypes (*n *=* *261). The average age at diagnosis was 72.

### EOC overall

**Figure 3.**
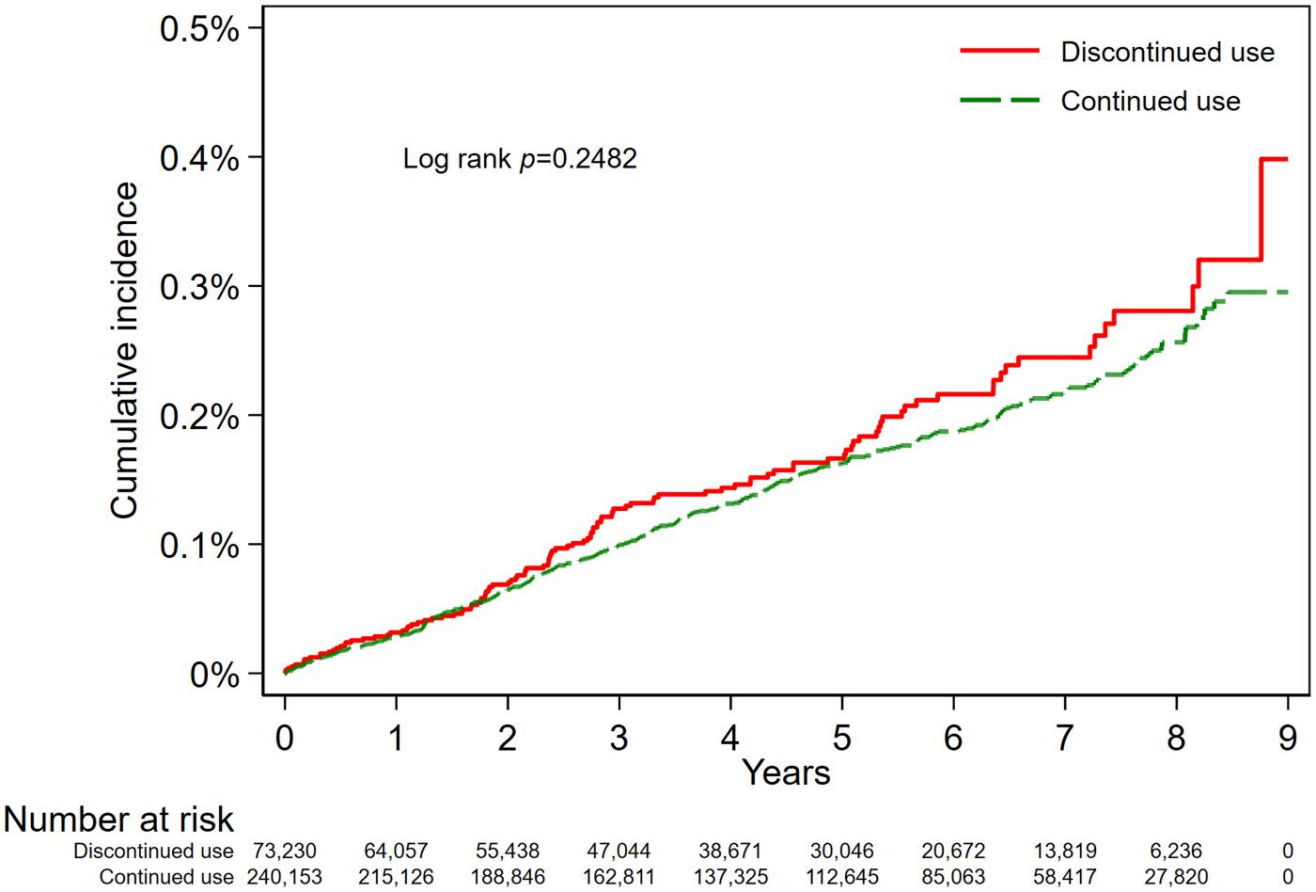
Kaplan–Meier cumulative incidence curve for outcome of epithelial ovarian cancer diagnosis in the discontinued and continued use groups

**Figure 4.**
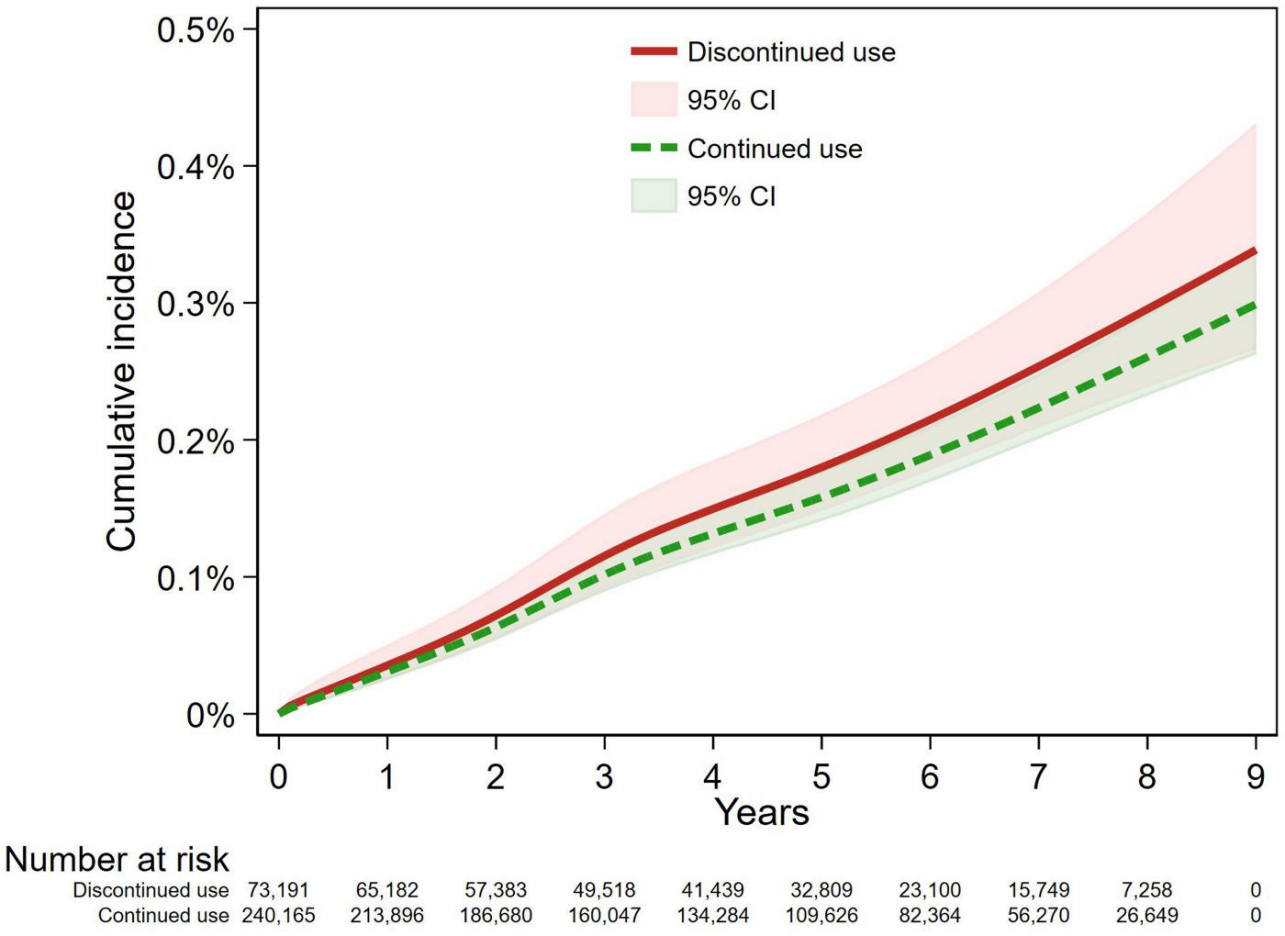
Inverse probability of treatment weighted model for outcome of epithelial ovarian cancer diagnosis for those women with discontinued use or continued use, and estimated 95% confidence intervals. CI, confidence interval

Table 2 shows the results of Models 1–3. Figures 3–4 show the Kaplan–Meier and inverse probability of treatment weighted flexible parametric cumulative incidence curves. There was a suggestive association between continued use of NBB and reduced risk of EOC overall, compared with discontinued treatment (Model 2: HR = 0.87, 95% CI: 0.69, 1.10). However, the flexible parametric curves showed that the confidence intervals of the two treatment groups largely overlapped (Figure 4). The HR for the association between continued NBB use and EOC diagnosis compared with discontinued NBB use remained below 1 throughout follow-up (Supplementary Figure S2, available as Supplementary data at *IJE* online). Use of inverse probability of time-varying treatment weights in Model 3 did not differ materially from Model 2 (HR = 0.89, 95% CI: 0.72, 1.10).

**Table 2.** Results for the association between continued treatment and risk of epithelial ovarian cancer overall and serous histotype

		Main: intention-to-treat			
			Model 1^a^	Model 2^b^	Model 3^c^
Treatment	*n*	Cases	HR (95% CI)	HR (95% CI)	HR (95% CI)
**EOC overall**					
Discontinued NBB use	73 230	114	Reference	Reference	Reference
Continued NBB use	240 153	358	0.88 (0.71, 1.09)	0.87 (0.69, 1.10)	0.89 (0.72, 1.10)
At each time point		C.Events			
6 months		56		0.91 (0.62, 1.35)	
1 year		86		0.91 (0.68, 1.22)	
2 years		183		0.88 (0.64, 1.21)	
3 years		274		0.87 (0.64, 1.19)	
4 years		329		0.88 (0.65, 1.18)	
5 years		376		0.88 (0.62, 1.26)	
6 years		414		0.88 (0.59, 1.34)	
7 years		441		0.89 (0.55, 1.42)	
8 years		461		0.89 (0.53, 1.49)	
9 years		472		0.89 (0.50, 1.57)	
**Serous EOC**					
Discontinued NBB use	73 230	74	Reference	Reference	Reference
Continued NBB use	240 153	187	0.71 (0.54, 0.93)	0.71 (0.53, 0.96)	0.72 (0.55, 0.95)
At each time point		C.Events			
6 months		28		0.71 (0.47, 1.08)	
1 year		47		0.71 (0.52, 0.98)	
2 years		100		0.72 (0.54, 0.94)	
3 years		158		0.72 (0.53, 0.97)	
4 years		179		0.72 (0.47, 1.09)	
5 years		204		0.72 (0.49, 1.06)	
6 years		226		0.72 (0.50, 1.03)	
7 years		241		0.72 (0.50, 1.04)	
8 years		254		0.72 (0.49, 1.06)	
9 years		261		0.72 (0.48, 1.09)	

C.Events, cumulative EOC diagnoses at each time point; CI, confidence interval; EOC, epithelial ovarian cancer; HR, hazard ratio; NBB, nitrogen-based bisphosphonates; SEIFA, Socio-Economic Indexes for Areas.

a Model 1: unweighted model adjusted for age at baseline.

b Model 2: flexible parametric survival models allowing for time-varying survival effects. Inverse probability of treatment weights were used to balance the treatment groups for all covariates as listed on Table 1, including age at first use, birth year, Medicare registered state, SEIFA quintile, remoteness category, Rx-Risk comorbidity categories and Pharmaceutical Benefits Scheme concessional status.

c Model 3: inverse probability of time-varying treatment weights (1-year time intervals) in a pooled logistic regression model, adjusting for pre-baseline covariates: age, menopausal hormone therapy use, ischaemic heart disease: angina, gastro-oesophageal reflux disease, steroid-responsive diseases and pain and time-varying covariates: menopausal hormone therapy use and another cancer diagnosis.

### Serous histotypes

**Figure 5.**
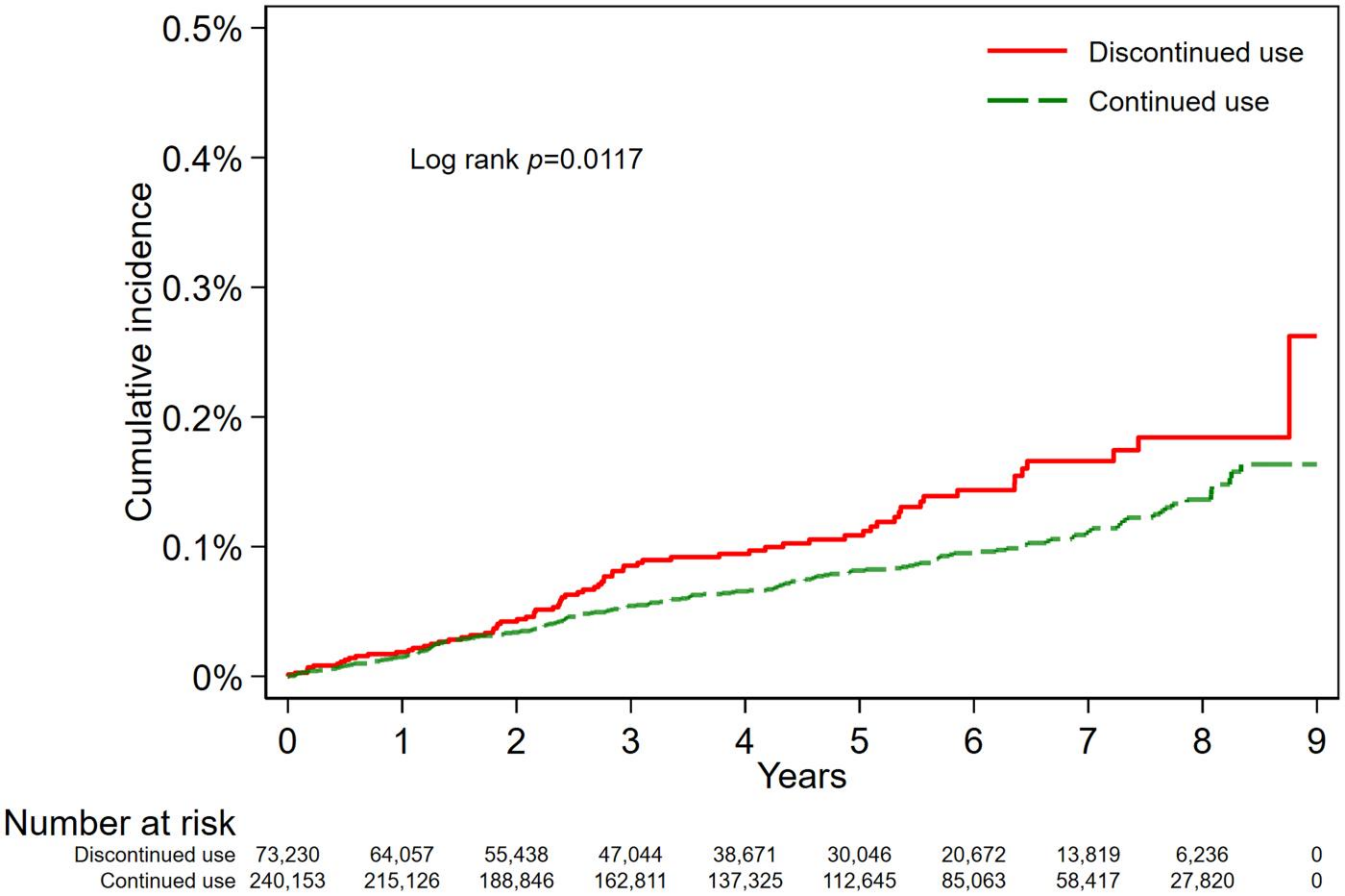
Kaplan–Meier cumulative incidence curve for outcome of serous histotype diagnosis in the discontinued and continued use groups

**Figure 6.**
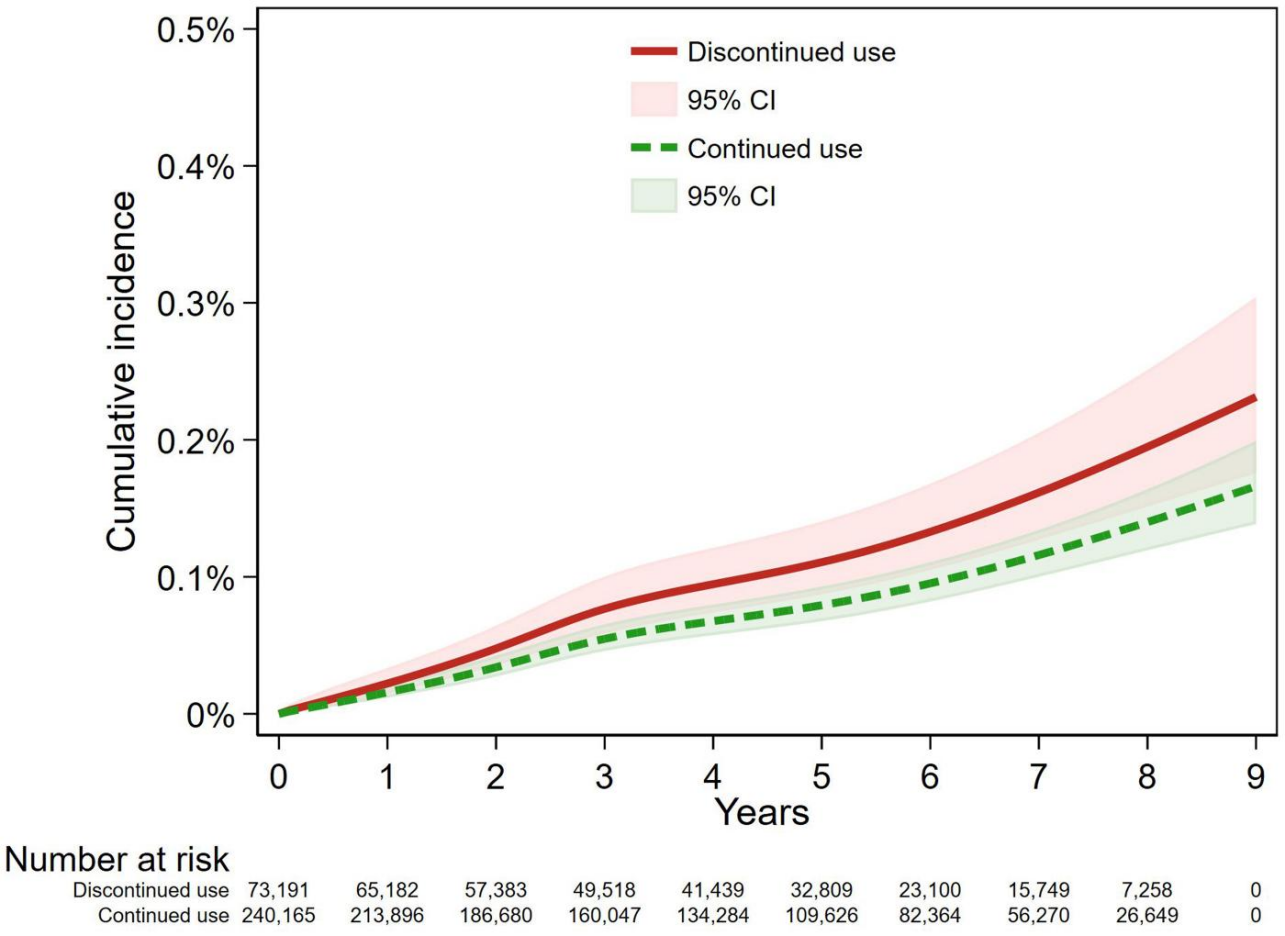
Inverse probability of treatment weighted model for outcome of serous EOC diagnosis for those women with discontinued use or continued use, and estimated 95% confidence intervals. CI, confidence interval; EOC, epithelial ovarian cancer

Continued use of NBB was associated with a reduced risk of serous EOC, compared with discontinued treatment (Model 2: HR = 0.71, 95% CI: 0.53, 0.96; Table 2, Figure 5–6). Supplementary Figure S3 (available as Supplementary data at *IJE* online), showing the HR over time, indicates that there was an association from as early as 1 year after the commencement of follow-up. The 95% confidence intervals included the null from around 3.5 years onwards, due to the reduced statistical power with smaller number of events, but the HR remained below 1. Model 3, using time-varying treatment weights, did not differ materially (HR = 0.72, 95% CI: 0.55, 0.95).

### Sensitivity analyses

Detailed results of the sensitivity analyses are included in the Supplementary Results, Tables S8–S12 and Figures S4–S24 (available as Supplementary data at *IJE* online). Per protocol analyses using inverse probability of censoring weights were not materially different from our ITT analyses for EOC overall (HR = 0.87, 95% CI: 0.69, 1.09) and serous EOC (HR = 0.70, 95% CI: 0.52, 0.93). However, the per protocol analyses using flexible parametric models showed a strengthening in the association over time (Supplementary Figures S4–S7, available as Supplementary data at *IJE* online). For women aged 70 years and younger at first NBB use, the estimates were slightly stronger, but the confidence intervals were wider. Overall, results of the sensitivity analyses did not differ materially from our main results. We did not find an association between continued NBB use and pancreatic cancer as a negative control cancer outcome (HR = 1.03, 95% CI: 0.85,1 .25, Supplementary Table S11, Figure S20, available as Supplementary data at *IJE* online).

## Discussion

Our emulated trial using Australian population-based data found that of women who initiated NBB treatment without a history of cancer, those who continued using NBBs had a 29% lower hazard of being diagnosed with serous EOC over 9 years of follow-up, compared with women who discontinued NBB use within 6 months of commencement. For EOC overall, hazard ratios indicated a reduced risk, but confidence intervals included 1.0.

Our previous nested case-control study found associations between ever use of NBBs and risk of the endometrioid and serous EOC histotypes, compared with no use (serous EOC: OR = 0.84,95% CI: 0.75, 0.93).^5^ However, it is possible that our earlier results were confounded by indication, because osteoporosis, which is associated with relatively low oestrogen levels, is the main indication for NBB use.^26^ In our current study, all women had an indication for NBB use, and whereas the study question is slightly different, our results have a much lower likelihood of confounding by indication, suggesting that our previously identified associations may not have been due to this confounding. It is still possible that there may be differences in women who discontinue use of NBBs after initiation; for example, they may have underlying conditions that increase the likelihood of side effects. However, we used inverse probability of treatment weights to balance the treatment groups for age, socioeconomic markers and pre-existing comorbidities that may increase the likelihood of having adverse side effects, including GORD, diabetes, hypertension, hyperthyroidism and pain.^27^ Our inverse probability weighted flexible parametric proportional hazards models produced similar estimates to the unweighted model, indicating that any imbalance between the treatment groups was not materially confounding the association between continued NBB use and the risk of EOC diagnosis. Furthermore, our per protocol sensitivity analyses suggested that the reduced risk of both EOC overall and serous EOC strengthened over time for women who continued to use NBB throughout follow-up.

There are biologically plausible explanations for our findings. NBBs have been shown to inhibit the mevalonate pathways within macrophages and monocytes, thereby reducing activity of tumour-associated macrophages and activating gamma delta T cells.^28^ These mechanisms potentially inhibit cancer cell proliferation, including in ovarian tumour cells.^29^^,^^30^ A study in mice treated with statins to inhibit the mevalonate pathway, showed a reduction in serous tubal intraepithelial carcinomas, a precursor to high-grade serous EOC.^31^ Further research is needed to understand how the mevalonate pathway may act on other EOC histotypes.

Our study had several strengths. Our emulated trial included over 300 000 women (240 153 and 73 230 per treatment group) with follow-up of up to 9 years, which would be difficult to achieve in an RCT. By including only women with an indication for NBB use, we reduced potential bias due to confounding for indication, and we used inverse probability of treatment weights to emulate randomization.^8^ Our study included all Australian women who first used NBBs during 2004–2012 at age 50 or older, and therefore our results are generalizable to Australian women with osteoporosis in this age group. Whereas the average age at baseline was 72 years, the results of our sensitivity including only women commencing use at 70 years or younger were not materially different. We used linked health records to ascertain medicine dispensing, rather than relying on self-report. We also had complete records of all cancers diagnosed for the women in the study.

Our study also had some limitations. Due to small numbers, we were unable to specifically assess the relationship between NBB use and histotypes other than serous EOC. It was possible for women to deviate from their treatment allocation during follow-up (40% of the continued and 20% of the discontinued treatment groups). This change in use could potentially bias the results in our ITT analysis towards the null. However our per protocol sensitivity analyses, where we censored women when they deviated from their treatment allocation, showed similar overall associations to the ITT analysis. There could be unmeasured confounding for other factors associated with both continuing NBB use and EOC, such as obesity or parity, but we used diabetes and hypertensive medicine use in our models, which are proxies for obesity. Whereas some low-cost medicines may not have complete capture in the PBS, such as MHT, rates of use in our cohort were sufficient to detect confounding effects if present. Our sensitivity analysis using a negative control cancer outcome (pancreatic cancer) which showed no association, indicates that our findings are unlikely to relate to unmeasured confounding that could increase the risk of cancers in general.

Conclusion

We used Australian population-based linked data to perform an emulated trial with the novel approach of assessing the impact of continued or discontinued NBB medication use by including only women indicated for use. Our results strengthen the existing evidence of the chemo-preventative potential of NBBs for EOC, especially for serous EOC, for which the results were stronger than in our previous nested case-control study that looked at ever versus never use. However, the results require replication.

## Ethics approval

The study was approved by the following human research ethics committees of the: University of Queensland, QIMR Berghofer Medical Research Institute, Australian Institute of Health and Welfare, Australian Capital Territory Health on 3 April 2017, New South Wales Population and Health Services, Western Australia Department of Health, Queensland Department of Health, Tasmania Health and Medical and Northern Territory Department of Health.

## Supplementary Material

dyae108_Supplementary_Data

## Data Availability

Because of the nature of this population-based research, participants of this study did not agree for their data to be shared publicly, so supporting data are not available.
