# Supplementary material for: Use of an emulated trial to investigate the association between use of nitrogen-based bisphosphonates and risk of epithelial ovarian cancer
Source: Int J Epidemiol. 2024 Aug 12;53(4):dyae108. doi: 10.1093/ije/dyae108 (PMC11319644; doi:10.1093/ije/dyae108)
Supplement: dyae108_Supplementary_Data [file dyae108_supplementary_data.pdf]

Supplementary Materials

This document contains supplementary materials for the manuscript:

"Use of an emulated trial to investigate the association between use of nitrogen-based bisphosphonates and risk of epithelial ovarian cancer."

Table of Contents

List of Supplementary Tables 2

List of Supplementary Figures 2

Supplementary Methods 6

Covariates 6

Pharmaceutical Benefits Scheme 6

Sensitivity analyses 7

Supplementary Results 10

Sensitivity analyses 10

References 48

List of Supplementary Tables

Supplementary Table S1: Emulated Trial Description 12
Supplementary Table S2: PBS item codes and defined daily doses for each medicine category. 14
Supplementary Table S3: CONCORD-2 defined ovarian cancer sites.[9] 15
Supplementary Table S4: Epithelial ovarian cancer histological groups and subtypes.[9] 16
Supplementary Table S5: Rx-Risk comorbidity categories, weights and ATC Codes included in the Rx-Risk comorbidity index.[5] 17
Supplementary Table S6: Characteristics of women excluded due to death or cancer diagnosis during treatment assignment. 18
Supplementary Table S7: Characteristics of treatment groups and standardised mean differences for unweighted and inverse probably of treatment weighted samples for Rx Risk comorbidities categories 19
Supplementary Table S8: Results for the association between continued treatment and risk of epithelial ovarian cancer overall and serous histotype in the per protocol sensitivity analyses (models S1.1-S1.4). 20
Supplementary Table S9: Results for the association between continued treatment and risk of epithelial ovarian cancer overall and serous histotype, sensitivity analyses excluding zoledronic acid (model S2.1), in women aged 70 years and younger (model S2.3) and using an alternative treatment definition (model S2.6). 21
Supplementary Table S10: Results for the association between continued treatment and risk of epithelial ovarian cancer overall and serous histotype, sensitivity analysis sensitivity analysis conditional on no diagnosis during the first year of follow-up (model S2.2). 22
Supplementary Table S11: Results for the association between continued treatment and risk of pancreatic cancer as a negative control cancer outcome (model S2.5). 23

List of Supplementary Figures

Supplementary Figure S1: Treatment group assignment and follow-up for intention to treat and per protocol. 24
Supplementary Figure S2: Hazard ratios and 95% confidence intervals over time for the association between continued nitrogen-based bisphosphonate use and epithelial ovarian cancer diagnosis, compared to discontinued use in the intention-to-treat model 25

Supplementary Figure S3: Hazard ratios and 95% confidence intervals over time for the association between continued nitrogen-based bisphosphonate use and serous ovarian cancer diagnosis, compared to discontinued use in the intention-to-treat model. 26
Supplementary Figure S4: Inverse probability weighted survival model for outcome of epithelial ovarian cancer diagnosis for those women with discontinued use or continued use and estimated 95% confidence intervals, per protocol sensitivity analysis (model S1.2)................... 27
Supplementary Figure S5: Hazard ratios and 95% confidence intervals over time for the association between continued nitrogen-based bisphosphonate use and epithelial ovarian cancer diagnosis, compared to discontinued use, per protocol sensitivity analysis (model S1.2). 28
Supplementary Figure S6: Inverse probability weighted survival model for outcome of serous ovarian cancer diagnosis for those women with discontinued use or continued use and estimated 95% confidence intervals, per protocol sensitivity analysis (model S1.2)................... 29
Supplementary Figure S7: Hazard ratios and 95% confidence intervals over time for the association between continued nitrogen-based bisphosphonate use and serous ovarian cancer diagnosis, compared to discontinued use, per protocol sensitivity analysis (model S1.2). 30
Supplementary Figure S8: Inverse probability weighted survival model for outcome of epithelial ovarian cancer diagnosis for those women with discontinued use or continued use and estimated 95% confidence intervals, sensitivity analysis with zoledronic acid users removed (model S2.1). 31
Supplementary Figure S9: Hazard ratios and 95% confidence intervals over time for the association between continued nitrogen-based bisphosphonate use and epithelial ovarian cancer diagnosis, compared to discontinued use, sensitivity analysis with zoledronic acid users removed (model S2.1)... 32
Supplementary Figure S10: Inverse probability weighted survival model for outcome of serous ovarian cancer diagnosis for those women with discontinued use or continued use and estimated 95% confidence intervals, sensitivity analysis with zoledronic acid users removed (model S2.1). 33
Supplementary Figure S11: Hazard ratios over time for the association between continued nitrogen-based bisphosphonate use and serous ovarian cancer diagnosis, compared to discontinued use, sensitivity analysis with zoledronic acid users removed (model S2.1). 34
Supplementary Figure S12: Inverse probability weighted survival model for outcome of epithelial ovarian cancer diagnosis for those women with discontinued use or continued use and

estimated 95% confidence intervals, sensitivity analysis conditional on no diagnosis during the first year of follow-up (model S2.2). 35
Supplementary Figure S13: Hazard ratios over time for the association between continued nitrogen-based bisphosphonate use and epithelial ovarian cancer diagnosis, compared to discontinued use, sensitivity analysis conditional on no diagnosis during the first year of follow-up (model S2.2). 36
Supplementary Figure S14: Inverse probability weighted survival model for outcome of serous ovarian cancer diagnosis for those women with discontinued use or continued use and estimated 95% confidence intervals, sensitivity analysis conditional on no diagnosis during the first year of follow-up (model S2.2). 37
Supplementary Figure S15: Hazard ratios over time for the association between continued nitrogen-based bisphosphonate use and serous ovarian cancer diagnosis, compared to discontinued use, sensitivity analysis conditional on no diagnosis during the first year of follow-up (model S2.2). 38
Supplementary Figure S16: Inverse probability weighted survival model for outcome of epithelial ovarian cancer diagnosis for those women with discontinued use or continued use and estimated 95% confidence intervals, sensitivity analysis for women aged 70 and younger at first use (model S2.3). 39
Supplementary Figure S17: Hazard ratios over time for the association between continued nitrogen-based bisphosphonate use and epithelial ovarian cancer diagnosis, compared to discontinued use, sensitivity analysis for women aged 70 and younger at first use (model S2.3). 40
Supplementary Figure S18: Inverse probability weighted survival model for outcome of serous ovarian cancer diagnosis for those women with discontinued use or continued use and estimated 95% confidence intervals, sensitivity analysis for women aged 70 and younger at first use (model S2.3). 41
Supplementary Figure S19: Hazard ratios over time for the association between continued nitrogen-based bisphosphonate use and serous ovarian cancer diagnosis, compared to discontinued use, sensitivity analysis for women aged 70 and younger at first use (model S2.3). 42
Supplementary Figure S20: Inverse probability weighted survival model for outcome of pancreatic cancer diagnosis for those women with discontinued use or continued use and estimated 95% confidence intervals (model S2.5). 43

Supplementary Figure S21: Inverse probability weighted survival model for outcome of epithelial ovarian cancer diagnosis for those women with discontinued use or continued use and estimated 95% confidence intervals, sensitivity analysis for alternative treatment definition – per protocol analysis (model S2.6). 44
Supplementary Figure S22: Hazard ratios over time for the association between continued nitrogen-based bisphosphonate use and epithelial ovarian cancer diagnosis, compared to discontinued use, sensitivity analysis for alternative treatment definition – per protocol analysis (model S2.6). 45
Supplementary Figure S23: Inverse probability weighted survival model for outcome of serous ovarian cancer diagnosis for those women with discontinued use or continued use and estimated 95% confidence intervals, sensitivity analysis for alternative treatment definition – per protocol analysis (model S2.6). 46
Supplementary Figure S24: Hazard ratios over time for the association between continued nitrogen-based bisphosphonate use and serous ovarian cancer diagnosis, compared to discontinued use, sensitivity analysis for alternative treatment definition (model S2.6)............ 47

Supplementary Methods

Covariates

We used postcode at Medicare enrolment to define state of residence, area-level socioeconomic status using the Socio-Economic Indexes for Areas (SEIFA) Index of Relative Socio-Economic Disadvantage,[1] and remoteness category using the Accessibility/Remoteness Index of Australia[2] (ARIA). We classified participants into SEIFA quintiles from the most disadvantaged (1st quintile) to the least (5th quintile) using the distribution for all women aged 18 years or older enrolled for Medicare in 2002. Remoteness categories included major cities, inner regional, outer regional, remote/very remote. We used the earliest available SEIFA (2001) and ARIA (2006) indices; however, if the relevant score was missing for a postcode, the 2006 or 2011 score was used.

Pharmaceutical Benefits Scheme

The Pharmaceutical Benefits Scheme (PBS) provides timely, reliable and affordable access to necessary medicines for Australians.[3] Under the PBS, the government subsidises the cost of medicine for most medical conditions. All Australian residents registered for Medicare are entitled to a subsidy under the PBS, however Australians eligible for a concession also receive a subsidy for low-cost medicines. Australians who hold a Pensioner concession card, Commonwealth Seniors Health Card, Health Care Card, or Department of Veterans' Affairs card are eligible for a concession. Co-payment (out-of-pocket cost) for concessional beneficiaries for each PBS medicine ranged from $3.60 to $5.90 in 2002 to 2013, with the remaining cost of the medicine paid for by the Australian Government. The co-payment for general beneficiaries, however, ranged from $22.40 to $36.10 during this time.[3] Prior to 2012, only dispensed medicines that attracted an Australian government subsidy were recorded on the PBS records. Our study includes records from the PBS, which includes all dispensed medicines that attracted an Australian government subsidy until 2012, and all dispensed PBS medicines after 2012. The cost of many medicines is above the co-payment amount for both general and concessional beneficiaries, and therefore the PBS records include most prescribed medicines from 2002 to 2013. All nitrogen-based bisphosphonates were above co-payment throughout our study period and therefore would be captured by PBS records for all women in our study.

We used PBS data to identify comorbidities at baseline using Rx-Risk comorbidity categories,[4] which has been mapped to Australian PBS item codes.[5] We were able to use the PBS data to ascertain most comorbidities included in the Rx-Risk score calculation. Some low-cost medicines

that fall under the government co-payment threshold may be under ascertained prior to 2012 for example, over-the-counter pain medications. However, over 80% of the women in the cohort were concessional beneficiaries during the study period, therefore medicines for most women will be captured in our analysis.

Sensitivity analyses

We performed several sensitivity analyses to explore the potential for bias in our main analyses.

1. Per protocol analysis

We performed per-protocol sensitivity analyses to assess the effect of non-adherence to the treatment allocation. The effect that NBB use may have on the risk of epithelial ovarian cancer (EOC) is likely to have a latency period, we designed our per protocol analyses to allow for a 12-month lag-period from non-adherence to censoring. For women in the continued use group, non-adherence was defined as 12 months of no dispensed prescriptions for NBBs. For women in the discontinued use, non-adherence occurred when a NBB prescription was dispensed during follow-up. We censored follow-up time 12 months after the date of non-adherence. Supplementary Figure S1 shows the treatment allocation and censoring times for the per protocol analysis.

After censoring, we replicated the models used for the intention-to-treat analyses, first in an unweighted Cox model adjusted for age (model S1.1) and then in a fitted flexible parametric model allowing for time-varying effects using inverse probability (IP) weights for treatment assignment (model S1.2). We then used IP of censoring weights (IPCW) in pooled logistic regression models[6] with one-year time intervals. Our IPCW models included pre-baseline variables: age, MHT use, and four comorbidities that had the greatest variation between treatment groups (ischaemic heart disease: angina, gastro-oesophageal reflux disease, steroid-responsive diseases and pain), and time-varying covariates: MHT use (during follow-up year) and another cancer diagnosis. We first used pooled logistic regression in an unweighted model adjusting for age (model 1.3) and then applying the IPCW in a doubly robust logistic regression model adjusting for age and the pre-baseline variables (model S1.4).

2. Other intention-to-treat (ITT) sensitivity analyses

We performed additional sensitivity analyses using the intention-to-treat survival models. These are described below.

a) Exclusion of zoledronic acid (model A2.1)

In this analysis, women who used zoledronic acid in the first 12 months after baseline were excluded, therefore, only women who used alendronate and risedronate were included. In our main ITT analysis, women with a single zoledronic acid injection during the first 12 months of use were categorised in the continued use group. By excluding these women in this sensitivity analysis, we could assess the extent any observed association might have been influenced by including women with a single injection of zoledronic acid in the continued use group.

b) Starting follow-up at two years after baseline (model S2.2)

We performed a sensitivity analysis conditional upon having no EOC diagnosis during the first year of follow-up. This was to assess whether there were any changes in our models when women diagnosed with EOC in the first year were excluded. In this analysis, follow-up commenced 12 months after treatment assignment, i.e. two years after the first NBB use.

c) Restricting to women aged 70 years and younger (model S2.3)

We performed a sensitivity analysis to only include women who initiated NBB use at 70 years or younger, to investigate whether our results were influenced by the older ages of women who commence use of bisphosphonates.

d) Death as a competing risk (model S2.4)

We repeated our ITT flexible parametric models with death as a competing event. For serous EOC as an outcome, we additionally included death and other EOC histotypes as competing events.

e) Pancreatic cancer as a negative control cancer outcome (model S2.5)

We explored the potential for bias due to unmeasured confounding for the association between use early use patterns of NBBs and future cancer diagnosis using a negative control cancer outcome. The purpose of this analysis was to investigate whether the same effect would be found for a cancer outcome that is not expected to be associated with NBB use. NBB use has been associated with other cancers, such as breast and other gynaecological cancers.[7] Therefore, we selected pancreatic cancer as the negative control outcome, as prior studies have not found an association between NBB use and risk of pancreatic cancer.[7]

f) Alternative treatment definition (model S2.6)

In our final sensitivity analysis, we used a different definition of continued NBB use. For this analysis, we excluded continuing users with 168 or less defined daily doses from the analysis. The definition of discontinued users did not change. We used both ITT and per protocol analyses for this model.

Supplementary Results

Sensitivity analyses

1. Per protocol analyses (models S1.1-S1.4)

In the per protocol sensitivity analyses, 20% of women in the discontinued use group recommenced use of NBBs during follow-up, while 40% of women in the continued use group stopped using NBBs for 12 or more months. Supplementary Table S8 shows the results for each of the per protocol sensitivity analyses. There were 379 women diagnosed with EOC overall and 210 with serous EOC after excluding those diagnosed after censoring. The results of the flexible parametric model with IP weighting for treatment allocation (model 1.2) showed slightly stronger associations with both reduced risk of EOC overall (HR=0.81, 95%CI:0.64,1.03) and serous EOC (HR=0.67, 95%CI:0.49,0.91) compared to our main ITT analyses. The inverse association, however, strengthened over the follow-up period, showing associations from four years onwards (three years for serous EOC) (Supplementary Table S8, Supplementary Figures S4-S7). These results show that women who continued to use NBBs during follow-up had a lower risk EOC overall after 4 years and lower risk of serous EOC after 3 years, compared to those who discontinued use and did not recommence use.

Unweighted pooled logistic regression models (model 1.3) produced slightly weaker estimates for the association between continued treatment and risk of EOC overall compared to the flexible parametric models (HR=0.84, 95% CI:0.67,1.06), however the results for serous EOC were consistent (HR=0.67, 95% CI:0.50,0.90). In the doubly robust pooled logistic regression models where we used IPCW (model S1.4), estimates were slightly weaker, however were not materially different from our main ITT analysis (EOC overall: HR=0.87, 95%CI:0.69,1.09; serous EOC: HR=0.70, 95%CI:0.52,0.93). Overall, estimates did not change materially for the different models used, and confidence intervals were largely overlapping.

2. ITT sensitivity analyses

a) Exclusion of zoledronic acid Exclusion of zoledronic acid (model S2.1)

For this sensitivity analysis, we excluded 4,702 women whose initial NBB use was zoledronic acid and 1729 women who used zoledronic acid within 12 months of baseline. Results, as presented in Supplementary Table S9 and Supplementary Figures S8-S11, did not differ appreciably from our main ITT analysis.

b) Conditional upon having no EOC diagnosis during the first year of follow-up (model S2.2)

In the sensitivity analysis conditional upon having no EOC diagnosis during the first year of follow-up, 64 and 22 EOC diagnoses were excluded from the continued and discontinued treatment groups, respectively. Adjusted results were slightly weaker for EOC overall (HR=0.91, 95%CI:0.70,1.18), but similar for serous EOC (HR=0.73, 95%CI:0.53,1.00) suggesting that the first year of follow-up was not strongly influencing our overall results (Supplementary Table S10, Supplementary Figures S12-S15).

c) Women aged 70 years and younger (model S2.3)

For the sensitivity analysis including only women commencing NBB use at 70 years or younger (n=128,633), estimates were slightly stronger, although confidence intervals were wider. (Supplementary Table S9, Supplementary Figures S16-S19).

d) Death as a competing risk (model S2.4)

There were 47,619 women who died during follow-up. When we repeated our analysis with death as a competing event (and other EOC histotypes for our analysis with serous EOC as the outcome) our results did not change materially for either EOC overall (HR=0.88, 95%CI: 0.71,1.09) or serous EOC (HR=0.72, 95%CI: 0.55,0.94). Continued use of NBBs was associated with a small inverse association with death, compared to discontinued users (HR=0.96, 95%CI: 0.94,0.98).

e) Negative control cancer outcome (model S2.5)

Our sensitivity analysis using a negative control cancer outcome showed no association between continued use of NBBs and risk of pancreatic cancer (HR=1.03, 95%CI:0.85,1.25), compared to discontinued use (Supplementary Table S12 and Supplementary Figure S20). There were 803 women diagnosed with pancreatic cancer during follow-up.

f) Alternative treatment definition (model S2.6)

For our final sensitivity analysis where we used an alternative definition for continued users (>168 daily doses), 17,279 women were excluded from the continued use group (Supplementary Table S9). For the ITT analysis, the overall results were not materially different from our main ITT analysis, however the association appeared to strengthen up to four years after follow-up commenced before weakening slightly. For the per protocol analysis (Supplementary Table S9 and Supplementary Figures S21-S24), however, the results were not materially different to our main per protocol analysis also using flexible parametric models (model 1.2).

Supplementary Table S1: Emulated Trial Description

**Analysis Plan component**	**Hypothetical Target Trial**	**Trial Emulation**
Eligibility Criteria	Australian women aged 50 years and older who had osteoporosis or a fragility fracture and were eligible for treatment with a nitrogen-based bisphosphonate (NBB) from January 2004 to December 2012.<br>Exclusions:<br>• Women with prior use of osteoporosis medicines from July 2002 to December 2003.<br>• Women with a cancer diagnosis prior to first use.	*As per target trial.*<br>Women were eligible 12 months after they were first dispensed a prescription for NBB from January 2004 to December 2012.
Treatment strategies	1. Discontinued use: NBB use for first 5 months, then replaced with a placebo.<br>2. Continued NBB use.	Treatment assigned at 12 months after initial NBB use (baseline).<br>1. Discontinued use:<br>• No more than 168 daily doses dispensed (6 months * 28 days), and<br>• No prescriptions dispensed after 6 months of initial dispensing.<br>2. Continued use: use of 6 months or more (daily doses and/or continued script dispensing) in the first 12 months.
Assignment procedures	Participants will be randomly assigned to either strategy at baseline and will be blinded to their treatment.	To emulate the random assignment of treatment strategies at baseline, inverse probability weighting for propensity for treatment will be applied using pre-baseline characteristics.
Follow-up period	Follow-up commences 12 months after first NBB use. Women who were diagnosed with cancer or who died prior to this time will be excluded. Follow-up ends at diagnosis of epithelial ovarian cancer, death, or end of follow-up (31 December 2013).	*As per target trial.*
Outcome	Epithelial ovarian cancer overall and serous histotypes. Cancers registered on the Australia Cancer Database.	*As per target trial.*

Analysis Plan component	Hypothetical Target Trial	Trial Emulation
Causal contrasts of interest	Our main analysis will be an intention-to-treat analysis: comparative effect of being assigned to the treatment strategies at the start of follow-up, regardless of whether the individuals continue following the strategies during follow-up.<br><br>Per-protocol sensitivity analysis: comparative effect of following the strategies specified in the study protocol. Women who deviate from treatment assignment are censored 1 year after non-adherence. Non-adherence defined as:<br>• For continued NBB use: 12 months of no use (therefore censored 2 years since last use)<br>• For discontinued NBB use: recommencement of NBB use (therefore censored 1 year after recommenced use.	*As per target trial.*
Analysis plan	Intention-to-treat: Analysis using flexible parametric survival model with time-varying effects. We will also apply a time-varying treatment weight (one-year intervals) in a pooled logistic regression model.<br><br>Per-Protocol: We will perform the flexible parametric models as per the intention-to-treat model. We will also use inverse probability of censoring weights in a pooled logistic regression model, using one-year time intervals for time-varying effects.	*As per target trial.*

NBB: nitrogen-based bisphosphonate

Supplementary Table S2: PBS item codes and defined daily doses for each medicine category.

Nitrogen-based bisphosphonate	ATC Code	PBS Item Code	DDD[8]
Alendronate	M05BB03	02194L	7
Alendronate	M05BA04	02215N	7
Alendronate	M05BB03	02224C	7
Alendronate	M05BB05	02273P	28
Alendronate	M05BA04	08090T	4
Alendronate	M05BA04	08102K	1
Alendronate	M05BA04	08511Y	7
Alendronate	M05BB03	09012H	7
Alendronate	M05BB03	09183H	7
Alendronate	M05BB05	09351E	28
Risedronate	M05BA07	02191H	7
Risedronate	M05BB02	02220W	28
Risedronate	M05BB04	02254P	28
Risedronate	M05BB02	04059P	28
Risedronate	M05BB04	04380M	28
Risedronate	M05BA07	04443W	1
Risedronate	M05BA07	04444X	7
Risedronate	M05BA07	08481J	1
Risedronate	M05BA07	08621R	7
Risedronate	M05BB02	08899J	28
Risedronate	M05BA07	08972F	7
Risedronate	M05BB02	08973G	28
Risedronate	M05BB04	08974H	28
Risedronate	M05BB04	09147K	28
Risedronate	M05BA07	09391G	30
Risedronate	M05BA07	08482K	6
Zoledronic Acid	M05BA08	06371H	1 course
Zoledronic Acid	M05BA08	09288W	1 course
Zoledronic Acid	M05BA08	09350D	1 course
Zoledronic Acid	M05BA08	09653C	1 course
Zoledronic Acid	M05BA08	10542W	1 course
Zoledronic Acid	M05BA08	10548E	1 course
Zoledronic Acid	M05BA08	10554L	1 course
Zoledronic Acid	M05BA08	10555M	1 course
Zoledronic Acid	M05BA08	10561W	1 course
Zoledronic Acid	M05BA08	10571J	1 course

ATC: Anatomical Therapeutic Chemical; DDD: defined daily dose; PBS: Pharmaceutical Benefits Scheme.

Supplementary Table S3: CONCORD-2 defined ovarian cancer sites.[9]

Topology	Description
C569	Malignant neoplasm of ovary
C482	Peritoneum
C570	Fallopian tube
C481	Specified parts of peritoneum
C579	Female genital tract, not of specific origin
C578	Overlapping lesion of female genital organs
C574	Uterine adnexa NOS
C480	Retroperitoneum
C571	Broad ligament

NOS: Not otherwise specified.

Supplementary Table S4: Epithelial ovarian cancer histological groups and subtypes.[9]

CONCORD-2 Morphology grouping	ICD-O morphology code
Type I epithelial	
Clear cell carcinoma	8005, 8310, 8443, 9110
Endometrioid carcinoma	8380, 8382-8383, 8560, 8570
Mucinous carcinoma	8470-8471, 8480-8482, 8490
Squamous carcinoma	8051-8084
Transitional cell or Brenner carcinoma	8120-8131, 9000
Type II epithelial	
Serous carcinoma	8050, 8441, 8450, 8460-8461
Mixed epithelial-stromal carcinoma	8313, 8323, 8381, 8930-8991, 9010-9030
Undifferentiated or other epithelial	8010-8015, 8020-8046, 8090-8110, 8140-8231, 8246-8300, 8311-8312, 8314-8322, 8324-8325, 8336-8337, 8341-8375, 8384-8440, 8452-8454, 8500-8551, 8561-8562, 8571-8589

ICD-O: International Classification of Diseases for Oncology

Supplementary Table S5: Rx-Risk comorbidity categories, weights and ATC Codes included in the Rx-Risk comorbidity index.[5]

Rx-Risk Comorbidity Category[a]	ATC Codes
Anticoagulants	B01AA03-B01AB06, B01AE07, B01AF01, B01AF02, B01AX05
Antiplatelets	B01AC04–B01AC30
Arrhythmia	C01AA05, C01BA01–C01BD01, C07AA07
Congestive heart failure	C03DA02–C03DA99, C07AB02 (if PBS item code is 8732N, 8733P, 8734Q, 8735R, 08818D), C07AB07, C07AG02, C07AB12, and both of (C03CA01–C03CC01) and (C09AA01–C09AX99, C09CA01–C09CX99)
Diabetes	A10AA01–A10BX99
GORD	A02BA01–A02BX05
Hyperlipidemia	A10BH03, C10AA01–C10BX09
Hypertension	C03AA01–C03BA11, C03DB01-C03DB99, C03EA01, C09BA02–C09BA09, C09DA02–C09DA08, C02AB01–C02AC05, C02DB02–C02DB99, (C03CA01–C03CCO1) or (C09CA01–C09CX99) but not both groups.
Hyperthyroidism	H03BA02, H03BB01
Hypothyroidism	H03AA01–H03AA02
IHD: angina	C01DA02–C01DA14, C01DX16, C08EX02
IHD: hypertension	C07AA01–C07AA06, C07AA08–C07AB03, C07AB02 (if PBS item code is not 8732N, 8733P, 8734Q, 8735R, 08818D) C07AG01, C08CA01–C08DB01, C09DB01–C09DB04, C09DX01, C09BB02–C09BB10, C09DX03, C10BX03
Inflammation/pain	M01AB01–M01AH06
Antineoplastic	L01AA01–L01XX41
Osteoporosis/Paget's[b]	M05BA01–M05BB05, M05BX03, M05BX04, G03XC01, H05AA02
Pain	N02AA01–N02AX02, N02AX06, N02AX52
Pulmonary hypertension	C02KX01–C02KX05, C02KX
Steroid-responsive disease	H02AB01–H02AB10
Transplant	L04AA01- L04AA21, L04AD01-L04AD02

ATC: Anatomical Therapeutic Chemical; GORD: gastro-oesophageal reflux disease; IHD: Ischaemic heart disease; PBS: Pharmaceutical Benefits Scheme.

[a] Includes combination medicines.

[b] Excluding nitrogen-based bisphosphonates per Supplementary Table S1.

Supplementary Table S6: Characteristics of women excluded due to death or cancer diagnosis during treatment assignment.

Characteristic	Death		Cancer	
Age (mean, standard deviation)	82 (9)		74 (10)	
Rx-Risk comorbidity categories (n, %)				
Anticoagulant medicines	2,596	28%	923	19%
Antiplatelet medicines	4,906	54%	1,595	33%
Arrhythmia medicines	2,386	26%	480	10%
Hypertension/pulmonary hypertension medicines	5,170	56%	2,404	50%
Hyperlipidaemia medicines	3,954	43%	2,221	46%
Hypothyroidism medicines	1,276	14%	518	11%
Ischaemic heart disease: angina medicines	2,603	28%	770	16%
Ischaemic heart disease: hypertension medicines	5,401	59%	2,163	45%
Congestive heart failure medicines	4,100	45%	1,405	29%
Antineoplastic medicines	508	6%	268	6%
Other osteoporosis medicines	208	2%	126	3%
Diabetes medicines	1,472	16%	572	12%
Pain medicines	5,807	63%	2,586	54%
Inflammation/pain medicines	5,165	56%	2,988	62%
Gastro-oesophageal reflux disease	5,652	62%	2,727	57%
Steroid-responsive disease	3,653	40%	1,588	33%
Transplant medicines	121	1%	47	1%
Hyperthyroidism medicines	200	2%	84	2%
Total	9,166		4,802	

Supplementary Table S7: Characteristics of treatment groups and standardised mean differences for unweighted and inverse probably of treatment weighted samples for Rx Risk comorbidities categories.

	Unweighted			IP Weighted		
	Discontinued use	Continued use	SMD[a]	Discontinued use	Continued use	SMD[a]
Characteristic	*n*=73,230	*n*=240,153		n^b=73,191.45	n^b=240,164.75	
Rx-Risk comorbidity categories:						
Anticoagulant medicines (%)	10,613 (14)	35,378 (15)	0.007	10,800 (15)	35,264 (15)	0.002
Antiplatelet medicines (%)	21,688 (30)	72,913 (30)	0.016	22,139 (30)	72,513 (30)	0.001
Arrhythmia medicines (%)	5,724 (8)	19,239 (8)	0.007	5,861 (8)	19,142 (8)	0.001
Hypertension medicines (%)	33,832 (46)	112,164 (47)	0.010	34,100 (47)	111,888 (47)	<0.001
Hyperlipidaemia medicines (%)	32,082 (44)	107,439 (45)	0.019	32,577 (45)	106,922 (45)	<0.001
Hypothyroidism medicines (%)	7,986 (11)	26,335 (11)	0.002	8,042 (11)	26,312 (11)	0.001
Ischaemic heart disease: angina medicines (%)	10,439 (14)	32,117 (13)	0.026	9,997 (14)	32,634 (14)	0.002
Ischaemic heart disease: hypertension medicines (%)	29,726 (41)	97,419 (41)	0.001	29,763 (41)	97,462 (41)	0.002
Congestive heart failure medicines (%)	17,391 (24)	57,861 (24)	0.008	17,629 (24)	57,688 (24)	0.002
Antineoplastic medicines (%)	2,833 (4)	10,373 (4)	0.023	3,103 (4)	10,126 (4)	0.001
Other osteoporosis medicines (%)	2,056 (3)	6,050 (3)	0.018	1,908 (3)	6,217 (3)	0.001
Diabetes medicines (%)	7,520 (10)	23,641 (10)	0.014	7,317 (10)	23,893 (10)	0.002
Pain medicines (%)	35,327 (48)	111,787 (47)	0.034	34,461 (47)	112,773 (47)	0.003
Inflammation/pain medicines (%)	43,583 (60)	144,538 (60)	0.014	43,909 (60)	144,160 (60)	0.001
Gastro-oesophageal reflux disease (%)	40,155 (55)	125,294 (52)	0.053	38,721 (53)	126,819 (53)	0.002
Steroid-responsive disease (%)	22,188 (30)	70,296 (29)	0.022	21,666 (30)	70.898 (30)	0.002
Transplant medicines (%)	915 (1)	3,003 (1)	<0.001	920 (1)	3,004 (1)	0.001
Hyperthyroidism medicines (%)	942 (1)	3,118 (1)	0.001	949 (1)	3111 (1)	<0.001
Pulmonary hypertension medicines (%)	12 (<1)	65 (<1)	<0.001	17 (<1)	59 (<1)	<0.001

IP: inverse probability; NBB: nitrogen-based bisphosphonate; SEIFA: Socio-Economic Indexes for Areas; SMD: standardised mean difference.

[a] A difference of <0.1 is generally considered acceptable.

[b] IP Weighted frequencies presented for *n*.

Supplementary Table S8: Results for the association between continued treatment and risk of epithelial ovarian cancer overall and serous histotype in the per protocol sensitivity analyses (models S1.1-S1.4).

		All EOC			Serous EOC		
	n	cases	model	HR (95% CI)	cases	model	HR (95% CI)
Discontinued NBB use	73,230	101		Reference	66		Reference
Continued NBB use	240,153	278	S1.1	0.79 (0.63,0.99)	144	S1.1	0.63 (0.47,0.84)
			S1.2	0.81 (0.64,1.03)		S1.2	0.67 (0.49,0.91)
Time point		CEvts			CEvts		
6 months		56		0.97 (0.66,1.44)	28		0.92 (0.55,1.54)
1 year		86		0.98 (0.72,1.35)	47		0.81 (0.55,1.20)
2 years		181		0.93 (0.66,1.31)	100		0.68 (0.44,1.06)
3 years		260		0.78 (0.57,1.06)	152		0.53 (0.34,0.82)
4 years		300		0.63 (0.45,0.87)	166		0.45 (0.28,0.72)
5 years		333		0.55 (0.36,0.83)	182		0.44 (0.26,0.76)
6 years		352		0.50 (0.30,0.83)	192		0.43 (0.23,0.80)
7 years		363		0.46 (0.26,0.84)	200		0.42 (0.21,0.83)
8 years		374		0.44 (0.23,0.84)	207		0.40 (0.19,0.86)
9 years		379		0.42 (0.21,0.84)	210		0.39 (0.18,0.88)
			S1.3	0.84 (0.67,1.06)		S1.3	0.67 (0.50,0.90)
			S1.4	0.87 (0.69,1.09)		S1.4	0.70 (0.52,0.93)

CEvts: cumulative events at each time point; CI: confidence interval; EOC: epithelial ovarian cancer; HR: hazard ratio; IPCW: inverse probably of censoring weights; NBB: nitrogen-based bisphosphonate.
S1.1: Unweighted Cox model adjusted for age.
S1.2: Fitted flexible parametric model allowing for time-varying effects using inverse probability weights for treatment assignment.
S1.3: Unweighted pooled logistic regression model adjusted for age.
S1.4: Pooled logistic regression model, weighted using IPCW, adjusting for age and pre-baseline variables (age, MHT use, ischaemic heart disease: angina, gastro-oesophageal reflux disease, steroid-responsive diseases and pain). IPCW models were time-varying at one-year intervals and included pre-baseline variables (age, MHT use, ischaemic heart disease: angina, gastro-oesophageal reflux disease, steroid-responsive diseases and pain) and time-varying covariates (MHT use and another cancer diagnosis).

Supplementary Table S9: Results for the association between continued treatment and risk of epithelial ovarian cancer overall and serous histotype, sensitivity analyses excluding zoledronic acid (model S2.1), in women aged 70 years and younger (model S2.3) and using an alternative treatment definition (model S2.6).

	Sensitivity: excl. zoledronic acid			Sensitivity: <=70 years			Sensitivity: Alternative treatment definition				
							Intention-to-treat			Per protocol	
	n	cases	HR (95% CI)	*n*	cases	HR (95% CI)	*n*	cases	HR (95% CI)	cases	HR (95% CI)
EOC Overall											
Discontinued NBB use	73,230	114	Reference	30,877	47	Reference	72,230	114	Reference	101	Reference
Continued NBB use [a]	233,722	350	0.87 (0.70,1.07)	97,756	138	0.81 (0.59,1.14)	222,874	326	0.86 (0.70,1.07)	256	0.78 (0.62,0.98)
Continued NBB use [b]			0.85 (0.67,1.07)			0.85 (0.59,1.22)			0.85 (0.68,1.08)		0.80 (0.63,1.02)
At each time point		CEvts			CEvts			CEvts		CEvts	
6 months		51	0.85 (0.57,1.25)		22	0.94 (0.51,1.76)		53	0.91 (0.62,1.35)	53	0.97 (0.65,1.45)
1 year		79	0.88 (0.65,1.17)		31	0.94 (0.60,1.49)		83	0.89 (0.66,1.20)	83	0.97 (0.71,1.34)
2 years		175	0.89 (0.65,1.22)		66	0.91 (0.54,1.53)		170	0.85 (0.62,1.17)	168	0.91 (0.65,1.28)
3 years		266	0.90 (0.65,1.23)		98	0.87 (0.51,1.49)		256	0.84 (0.61,1.15)	245	0.77 (0.57,1.05)
4 years		321	0.89 (0.66,1.20)		115	0.80 (0.51,1.27)		307	0.85 (0.63,1.16)	283	0.62 (0.45,0.87)
5 years		368	0.88 (0.61,1.26)		136	0.76 (0.47,1.22)		349	0.87 (0.60,1.25)	314	0.55 (0.36,0.84)
6 years		406	0.87 (0.58,1.31)		154	0.75 (0.44,1.28)		384	0.88 (0.58,1.33)	330	0.50 (0.30,0.84)
7 years		433	0.87 (0.54,1.39)		166	0.73 (0.40,1.35)		410	0.88 (0.55,1.41)	341	0.47 (0.26,0.85)
8 years		453	0.86 (0.51,1.46)		179	0.72 (0.36,1.43)		429	0.89 (0.53,1.49)	352	0.44 (0.23,0.85)
9 years		464	0.86 (0.49,1.53)		185	0.73 (0.33,1.51)		440	0.89 (0.51,1.57)	357	0.42 (0.21,0.86)
Serous EOC											
Discontinued NBB use	73,230	74	Reference	30,877	38	Reference	72,230	74		66	
Continued NBB use [a]	233,722	182	0.70 (0.53,0.91)	97,756	86	0.63 (0.43,0.92)	222,874	169	0.69 (0.53,0.91)	133	0.62 (0.46,0.84)
Continued NBB use [b]			0.70 (0.52,0.93)			0.67 (0.44,1.02)			0.71 (0.53,0.96)		0.67 (0.49,0.91)
At each time point		CEvts			CEvts			CEvts		CEvts	
6 months		26	0.68 (0.45,1.04)		14	0.70 (0.37,1.30)		28	0.91 (0.54,1.55)	28	0.94 (0.55,1.58)
1 year		43	0.69 (0.50,0.96)		18	0.68 (0.42,1.10)		47	0.76 (0.52,1.12)	47	0.81 (0.54,1.20)
2 years		95	0.70 (0.53,0.92)		41	0.67 (0.45,0.98)		93	0.60 (0.40,0.90)	93	0.67 (0.44,1.03)
3 years		153	0.71 (0.53,0.96)		64	0.65 (0.44,0.97)		148	0.56 (0.37,0.86)	144	0.52 (0.34,0.80)
4 years		174	0.73 (0.48,1.10)		75	0.64 (0.40,1.02)		168	0.64 (0.41,1.00)	158	0.45 (0.28,0.72)
5 years		199	0.72 (0.49,1.07)		89	0.64 (0.39,1.05)		191	0.74 (0.45,1.22)	174	0.44 (0.25,0.76)
6 years		221	0.72 (0.50,1.04)		102	0.64 (0.39,1.04)		210	0.78 (0.47,1.28)	181	0.43 (0.22,0.81)
7 years		236	0.72 (0.50,1.04)		110	0.64 (0.39,1.05)		224	0.81 (0.47,1.38)	189	0.41 (0.20,0.85)
8 years		249	0.72 (0.49,1.06)		119	0.64 (0.38,1.07)		236	0.83 (0.46,1.50)	196	0.40 (0.18,0.88)
9 years		256	0.72 (0.48,1.08)		124	0.63 (0.37,1.10)		243	0.85 (0.45,1.62)	199	0.39 (0.17,0.91)

CEvts: cumulative events at each time point; CI: confidence interval; EOC: epithelial ovarian cancer; HR: hazard ratio; NBB: nitrogen-based bisphosphonates; PBS: Pharmaceutical Benefits Scheme; SEIFA: Socio-Economic Indexes for Areas.

[a] Unweighted model adjusted for age at baseline.

[b] Flexible parametric survival models allowing for time-varying survival effects. Inverse probability weights were used to balance the treatment groups for all covariates as listed on Table 1 including age at first use, birth year, Medicare registered state, SEIFA quintile, remoteness category, Rx-Risk comorbidity categories, and PBS concessional status.

Supplementary Table S10: Results for the association between continued treatment and risk of epithelial ovarian cancer overall and serous histotype, sensitivity analysis sensitivity analysis conditional on no diagnosis during the first year of follow-up (model S2.2).

	n	cases	HR (95% CI)
EOC Overall			
Discontinued NBB use	64,054	92	Reference
Continued NBB use [a]	215,108	294	0.88 (0.70,1.12)
Continued NBB use [b]			0.91 (0.70,1.18)
At each time point [c]		CEvts	
6 months		49	0.80 (0.50,1.27)
1 year		97	0.80 (0.55,1.16)
2 years		188	0.91 (0.62,1.34)
3 years		243	0.92 (0.64,1.31)
4 years		290	0.87 (0.61,1.25)
5 years		328	0.85 (0.56,1.29)
6 years		355	0.83 (0.51,1.36)
7 years		375	0.82 (0.47,1.44)
8 years		386	0.82 (0.44,1.51)
Serous EOC			
Discontinued NBB use	64,054	61	Reference
Continued NBB use [a]	215,108	153	0.69 (0.52,0.94)
Continued NBB use [b]			0.73 (0.53,1.00)
At each time point [c]		CEvts	
6 months		34	0.78 (0.51,1.18)
1 year		53	0.72 (0.53,0.99)
2 years		111	0.68 (0.50,0.92)
3 years		132	0.63 (0.44,0.92)
4 years		157	0.63 (0.43,0.93)
5 years		179	0.64 (0.44,0.92)
6 years		194	0.63 (0.43,0.93)
7 years		207	0.63 (0.42,0.93)
8 years		214	0.62 (0.41,0.94)

CEvts: cumulative events at each time point; CI: confidence interval; EOC: epithelial ovarian cancer; HR: hazard ratio; NBB: nitrogen-based bisphosphonates; PBS: Pharmaceutical Benefits Scheme; SEIFA: Socio-Economic Indexes for Areas.

[a] Model 1: Unadjusted model.

[b] Model 2: Flexible parametric survival models allowing for time-varying survival effects. Inverse probability weights were used to balance the treatment groups for all covariates as listed on Table 1 including age at first use, birth year, Medicare registered state, SEIFA quintile, remoteness category, Rx-Risk comorbidity categories, and PBS concessional status.

[c] Follow-up commenced 12 months after treatment assignment, which was 24 months after first use of an NBB.

Supplementary Table S11: Results for the association between continued treatment and risk of pancreatic cancer as a negative control cancer outcome (model S2.5).

Comparator	*n*	cases	HR (95% CI)
Discontinued NBB use	73,230	170	Reference
Continued NBB use [a]	233,722	633	1.03 (0.87,1.22)
Continued NBB use [b]			1.03 (0.85,1.25)

CI: confidence interval; HR: hazard ratio; NBB: nitrogen-based bisphosphonates; PBS: Pharmaceutical Benefits Scheme; SEIFA: Socio-Economic Indexes for Areas.

[a] Unweighted model adjusted for age at baseline.

[b] Flexible parametric survival models allowing for time-varying survival effects. Inverse probability weights were used to balance the treatment groups for all covariates as listed on Table 1 including age at first use, birth year, Medicare registered state, SEIFA quintile, remoteness category, Rx-Risk comorbidity categories, and PBS concessional status.

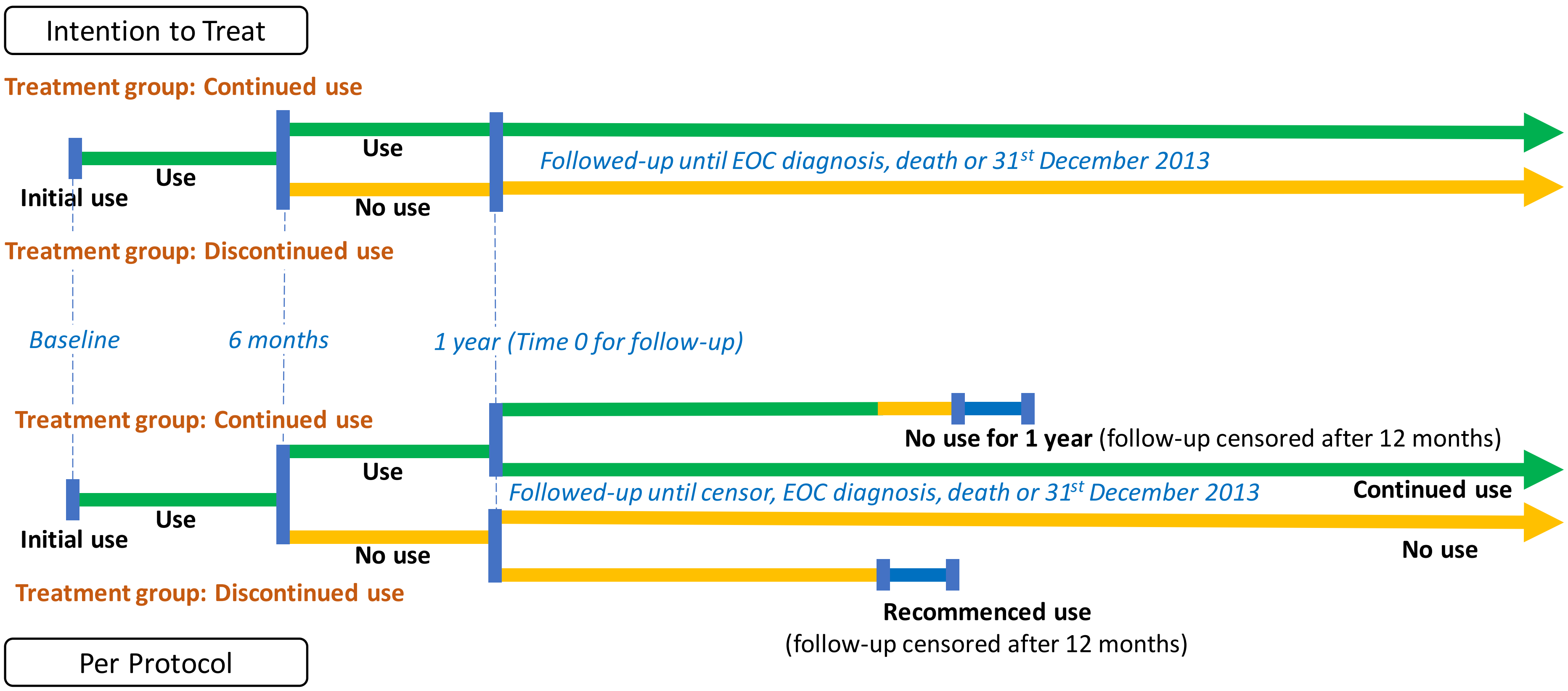

<u>Supplementary Figure S1: Treatment group assignment and follow-up for intention to treat and per protocol.</u>

EOC: epithelial ovarian cancer.

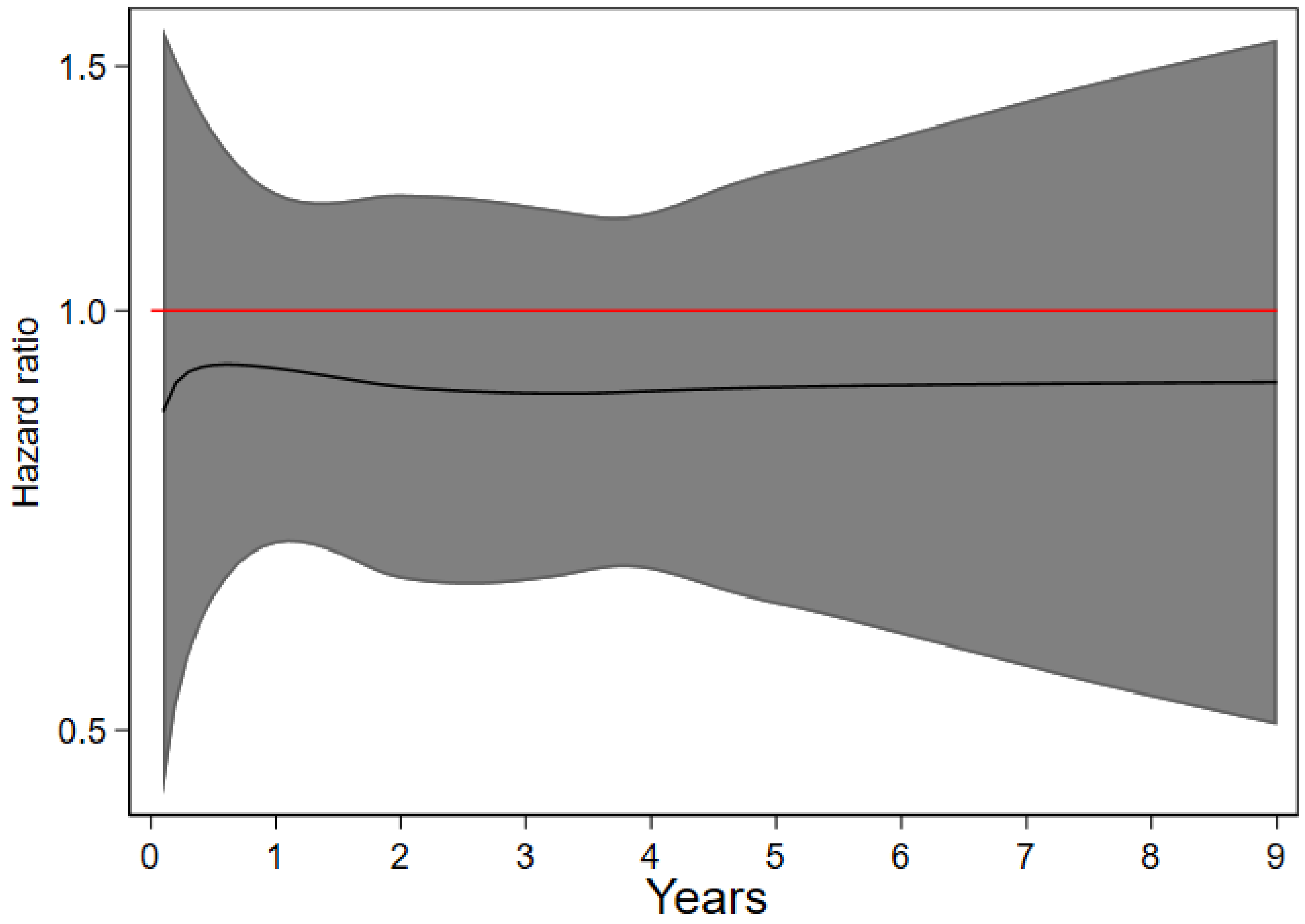

Supplementary Figure S2: Hazard ratios and 95% confidence intervals over time for the association between continued nitrogen-based bisphosphonate use and epithelial ovarian cancer diagnosis, compared to discontinued use in the intention-to-treat model.

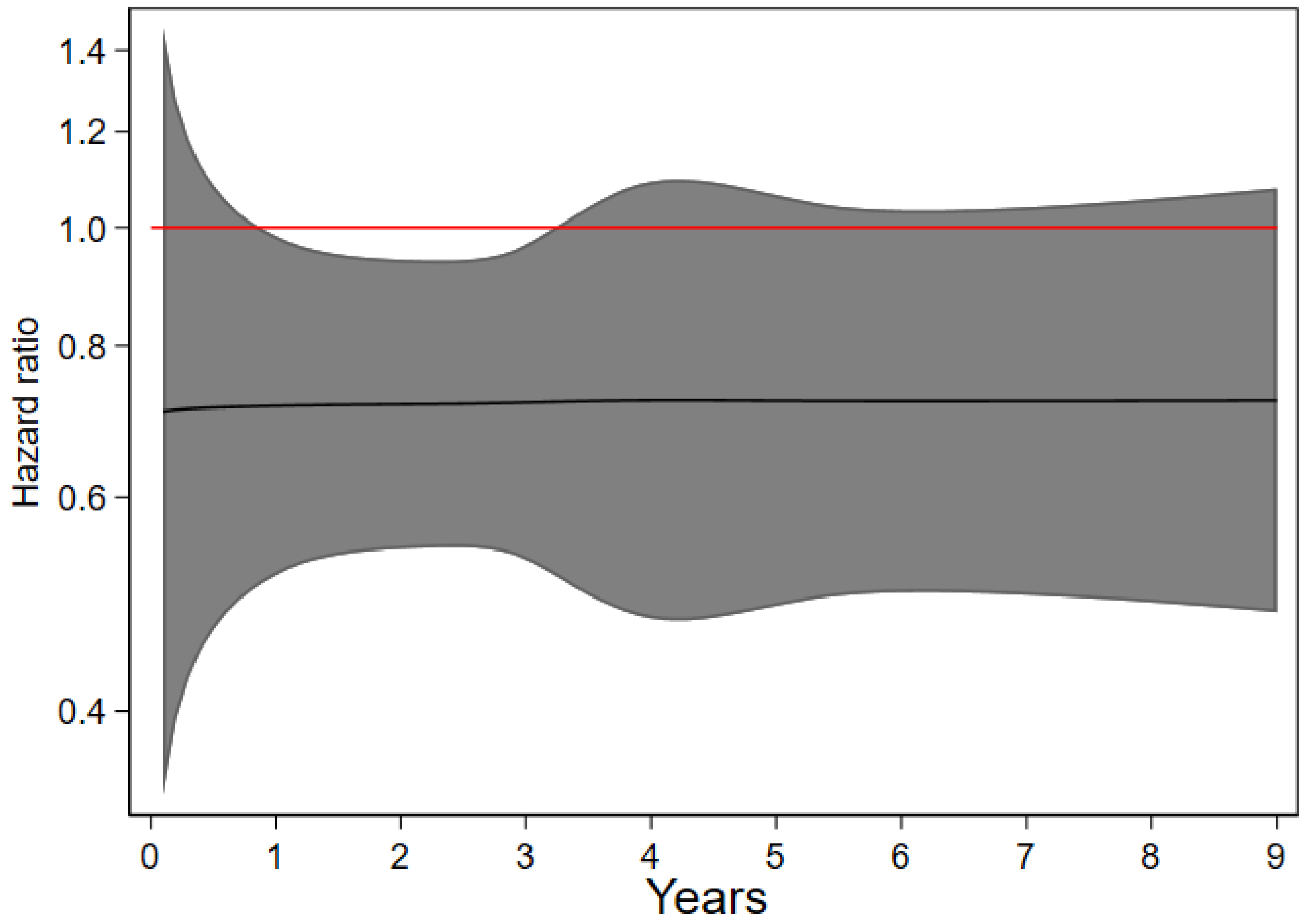

Supplementary Figure S3: Hazard ratios and 95% confidence intervals over time for the association between continued nitrogen-based bisphosphonate use and serous ovarian cancer diagnosis, compared to discontinued use in the intention-to-treat model.

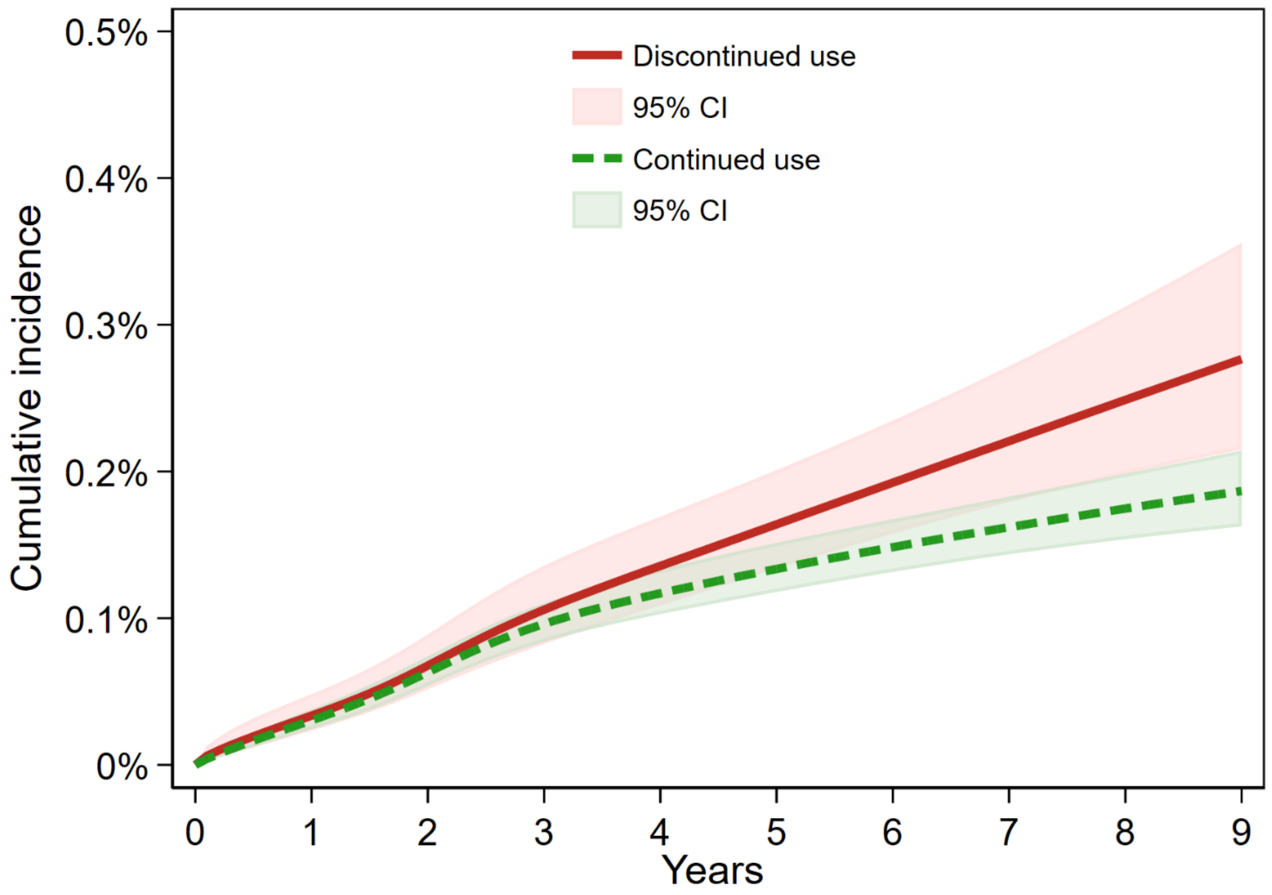

Number at risk

	0	1	2	3	4	5	6	7	8	9
Discontinued use	73,191	65,182	57,383	49,518	41,439	32,809	23,100	15,749	7,258	0
Continued use	240,165	213,896	186,680	160,047	134,284	109,626	82,364	56,270	26,649	0

Supplementary Figure S4: Inverse probability weighted survival model for outcome of epithelial ovarian cancer diagnosis for those women with discontinued use or continued use and estimated 95% confidence intervals, per protocol sensitivity analysis (model S1.2).

CI: confidence interval

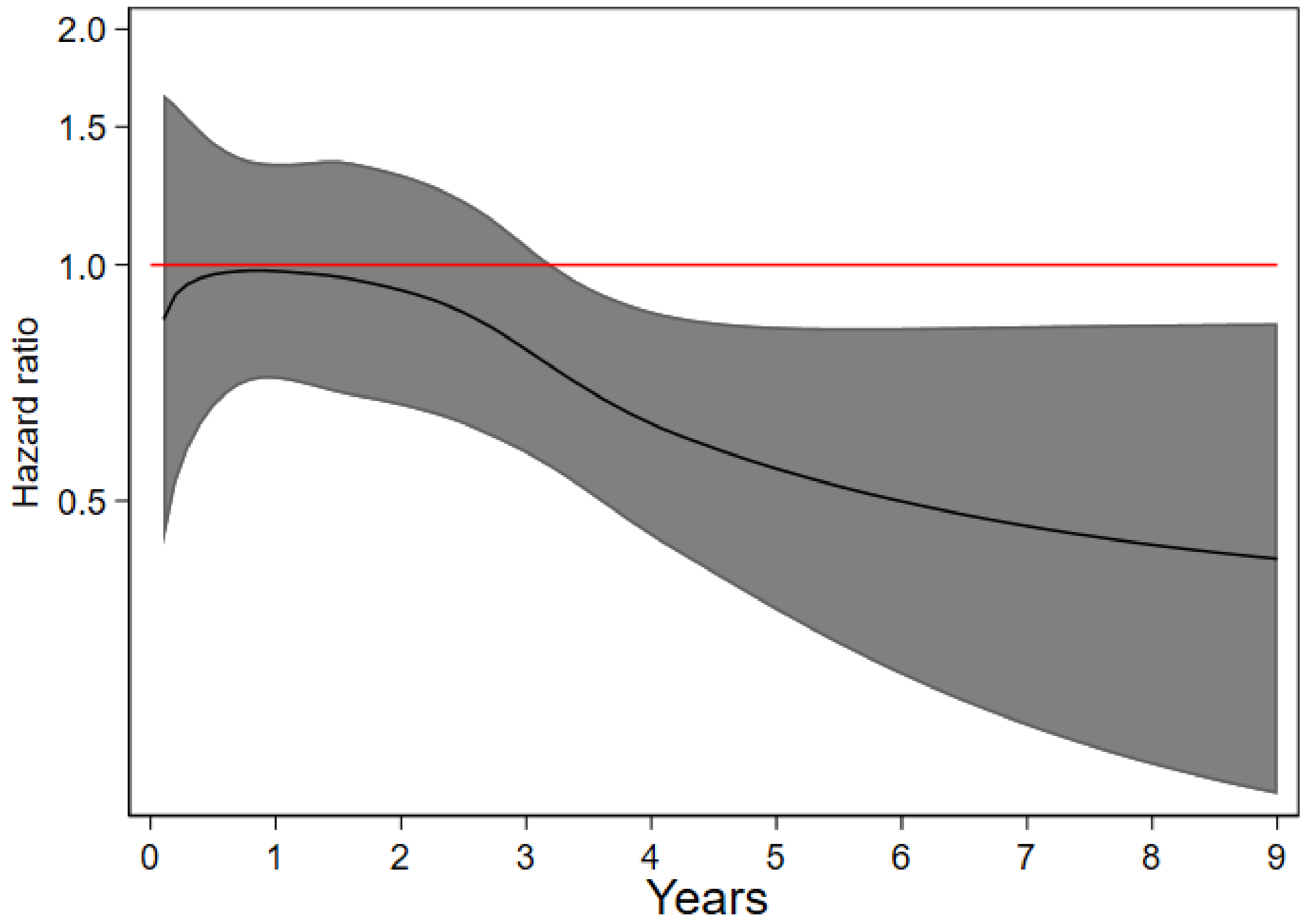

Supplementary Figure S5: Hazard ratios and 95% confidence intervals over time for the association between continued nitrogen-based bisphosphonate use and epithelial ovarian cancer diagnosis, compared to discontinued use, per protocol sensitivity analysis (model S1.2).

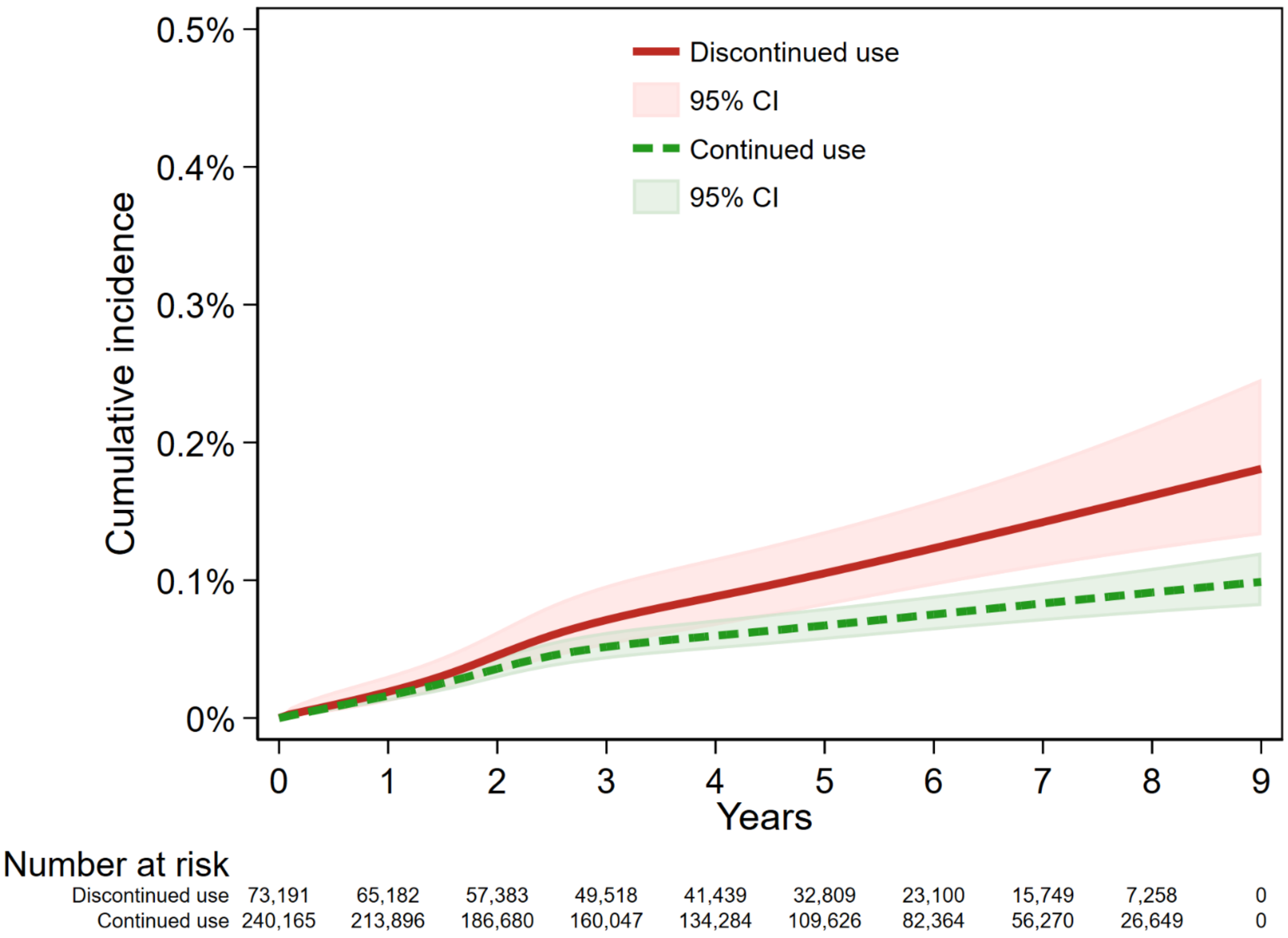

Supplementary Figure S6: Inverse probability weighted survival model for outcome of serous ovarian cancer diagnosis for those women with discontinued use or continued use and estimated 95% confidence intervals, per protocol sensitivity analysis (model S1.2).

CI: confidence interval.

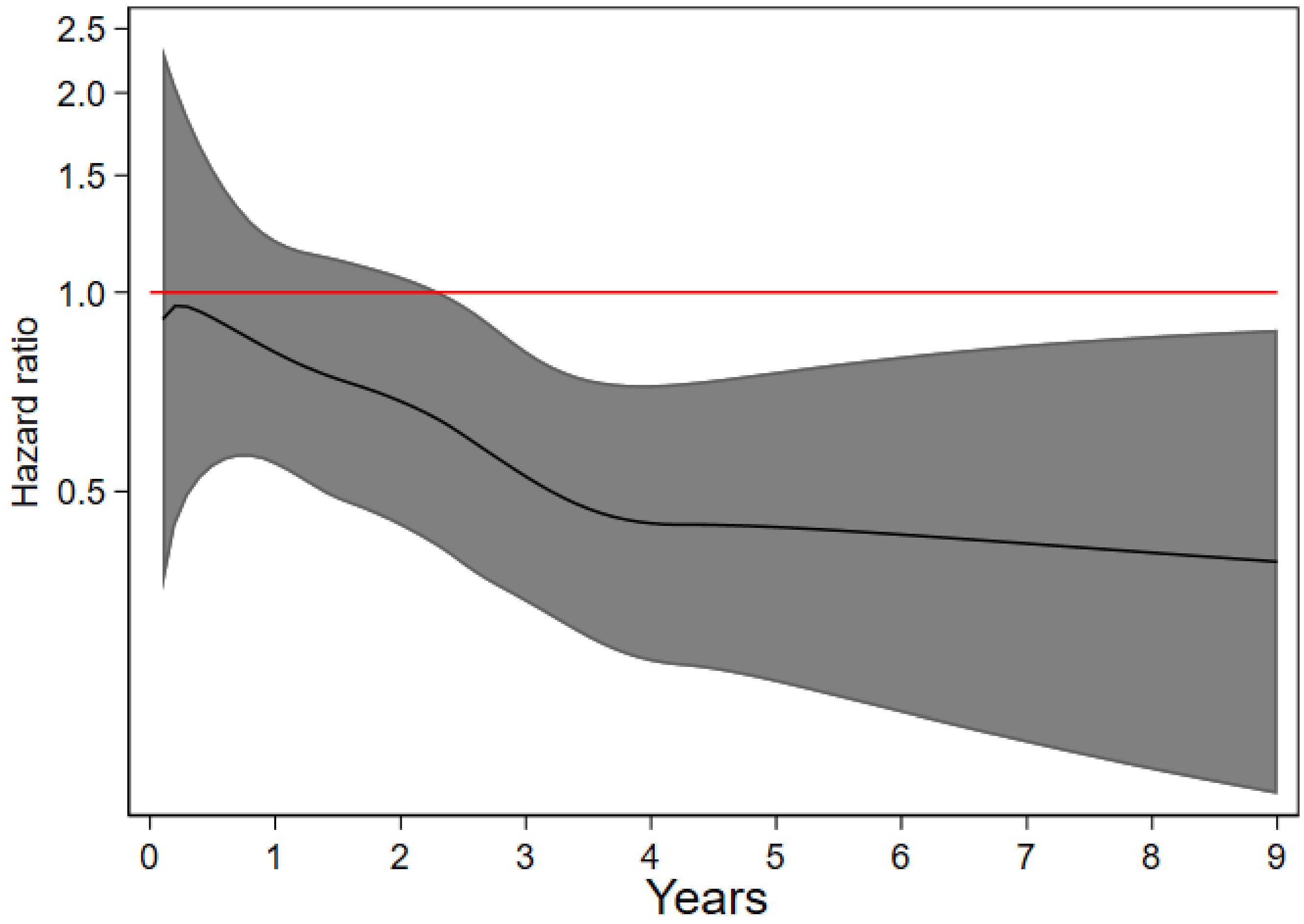

Supplementary Figure S7: Hazard ratios and 95% confidence intervals over time for the association between continued nitrogen-based bisphosphonate use and serous ovarian cancer diagnosis, compared to discontinued use, per protocol sensitivity analysis (model S1.2).

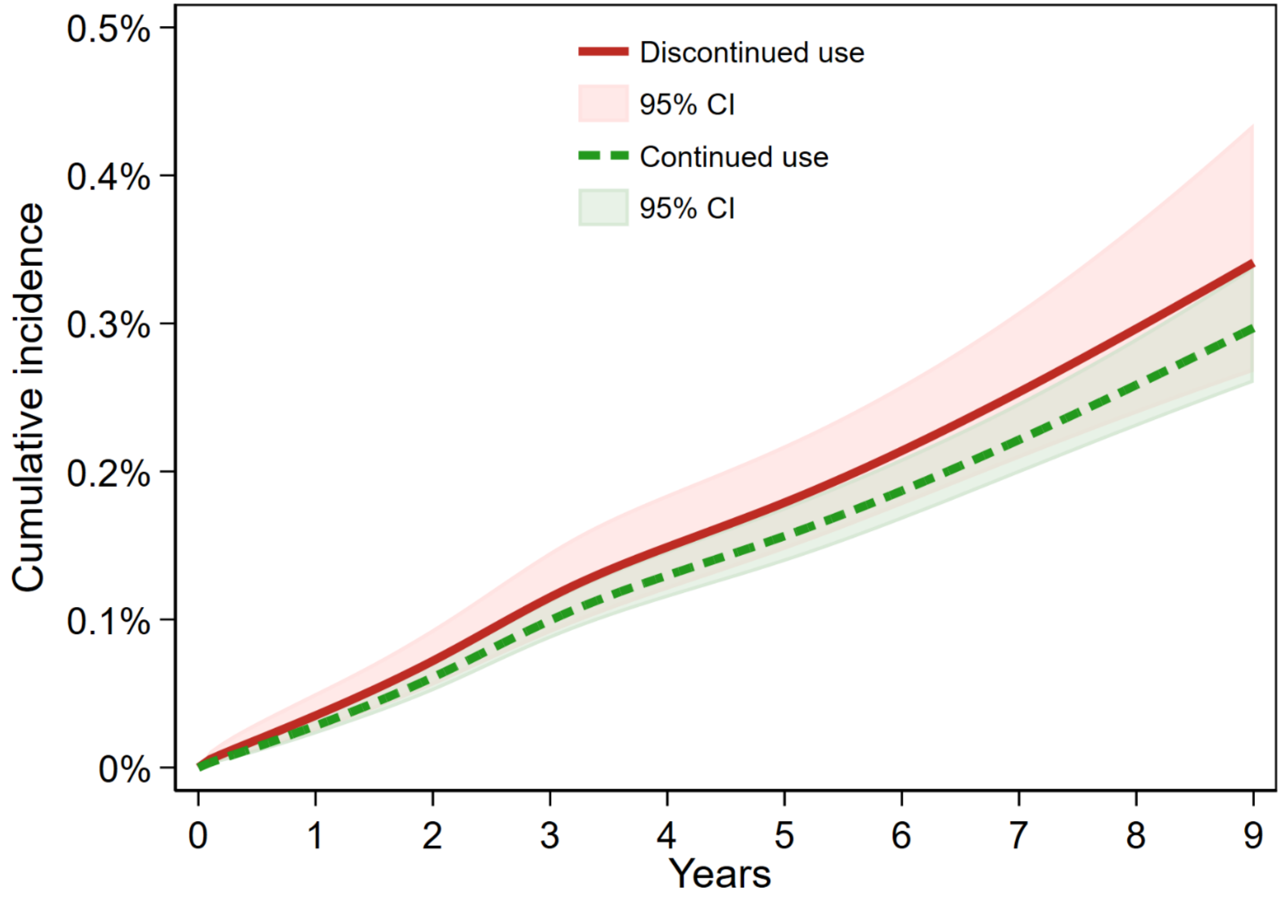

Number at risk

Discontinued use	73,182	65,455	57,869	50,155	42,173	33,565	23,785	16,300	7,557	0
Continued use	233,737	209,885	183,987	158,468	133,408	108,832	81,648	55,708	26,345	0

Supplementary Figure S8: Inverse probability weighted survival model for outcome of epithelial ovarian cancer diagnosis for those women with discontinued use or continued use and estimated 95% confidence intervals, sensitivity analysis with zoledronic acid users removed (model S2.1).

CI: confidence interval

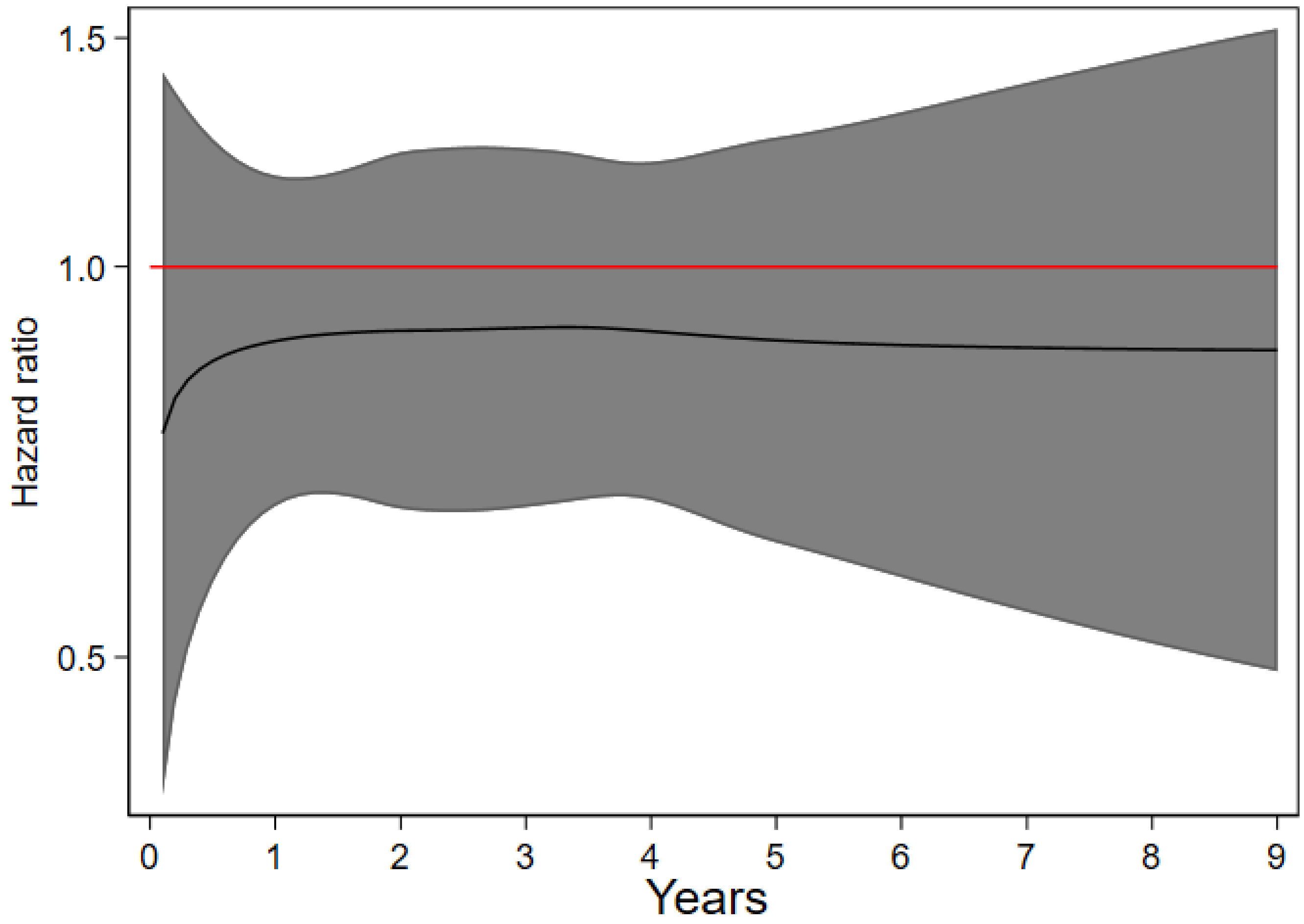

Supplementary Figure S9: Hazard ratios and 95% confidence intervals over time for the association between continued nitrogen-based bisphosphonate use and epithelial ovarian cancer diagnosis, compared to discontinued use, sensitivity analysis with zoledronic acid users removed (model S2.1).

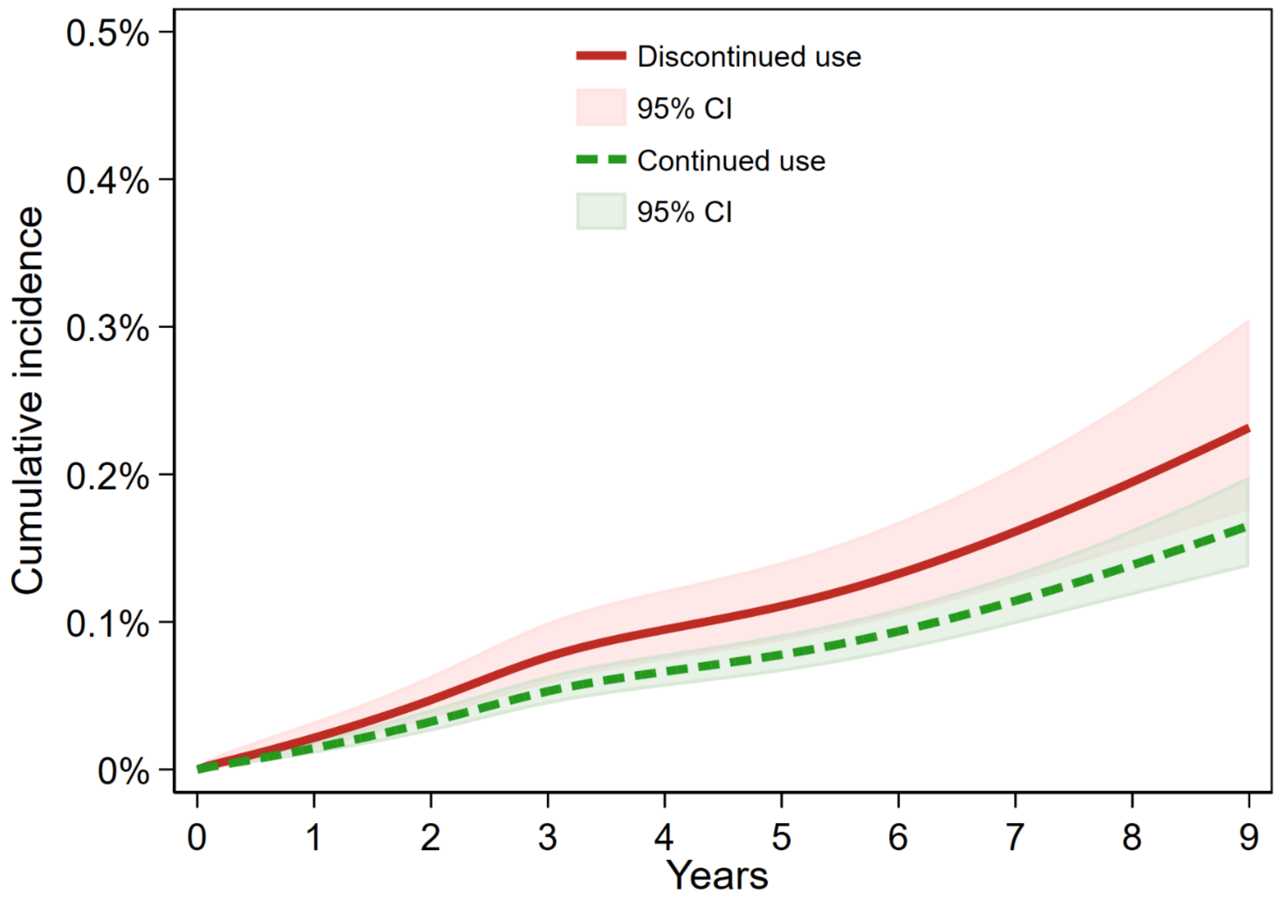

Number at risk

	0	1	2	3	4	5	6	7	8	9
Discontinued use	73,182	65,455	57,869	50,155	42,173	33,565	23,785	16,300	7,557	0
Continued use	233,737	209,885	183,987	158,468	133,408	108,832	81,648	55,708	26,345	0

Supplementary Figure S10: Inverse probability weighted survival model for outcome of serous ovarian cancer diagnosis for those women with discontinued use or continued use and estimated 95% confidence intervals, sensitivity analysis with zoledronic acid users removed (model S2.1).

CI: confidence interval

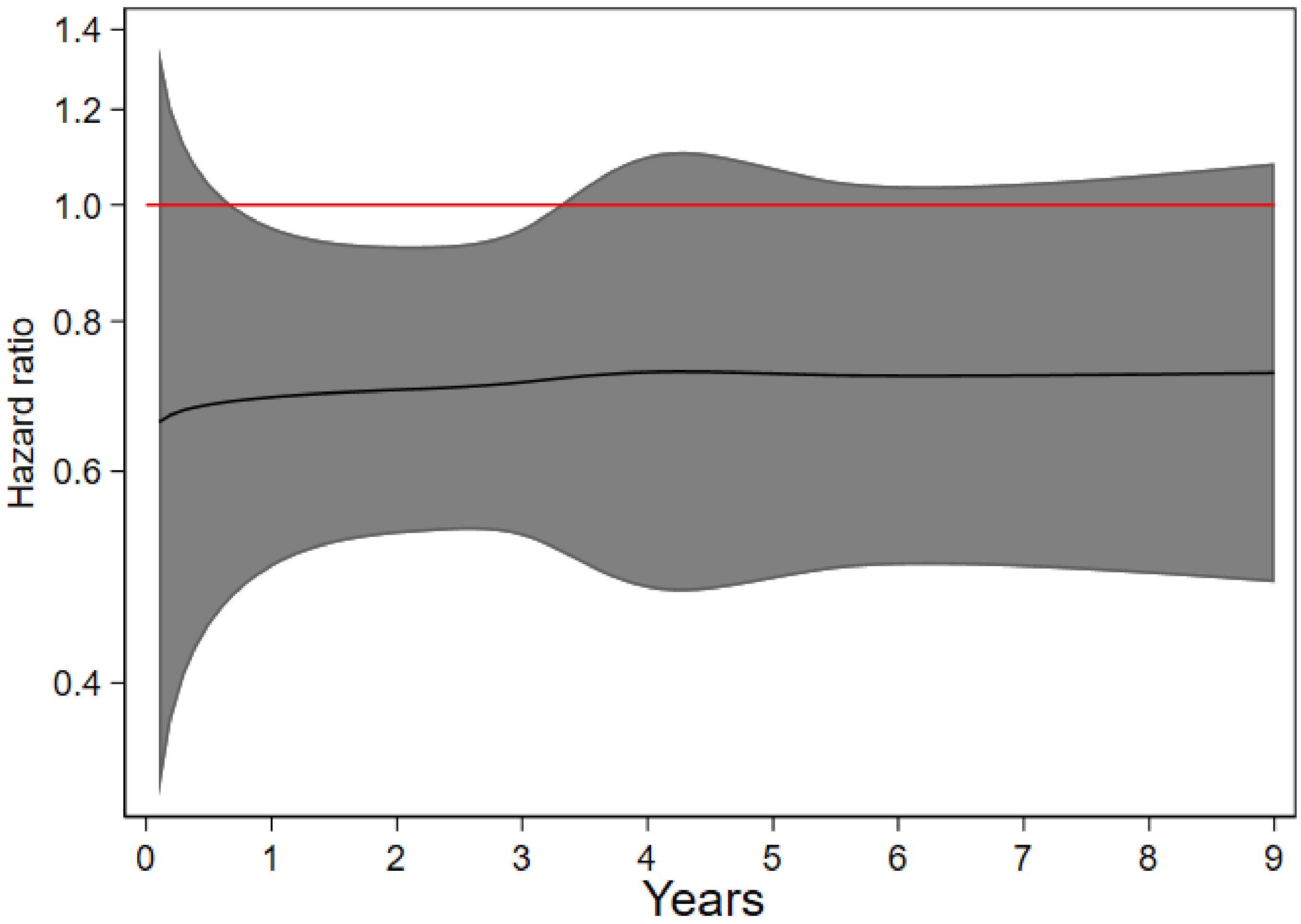

Supplementary Figure S11: Hazard ratios over time for the association between continued nitrogen-based bisphosphonate use and serous ovarian cancer diagnosis, compared to discontinued use, sensitivity analysis with zoledronic acid users removed (model S2.1).

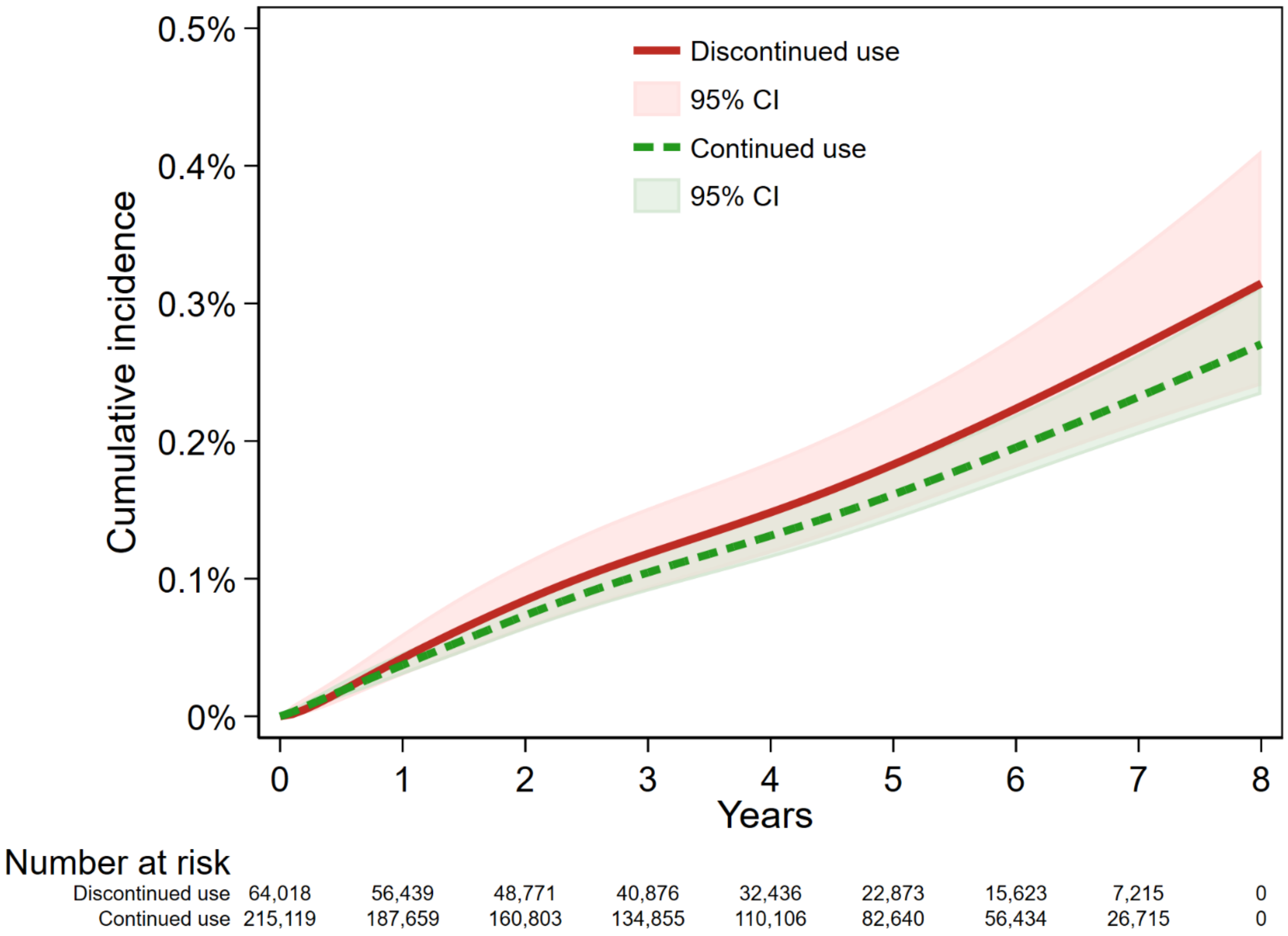

<u>Supplementary Figure S12: Inverse probability weighted survival model for outcome of epithelial ovarian cancer diagnosis for those women with discontinued use or continued use and estimated 95% confidence intervals, sensitivity analysis conditional on no diagnosis during the first year of follow-up (model S2.2).</u>

CI: confidence interval

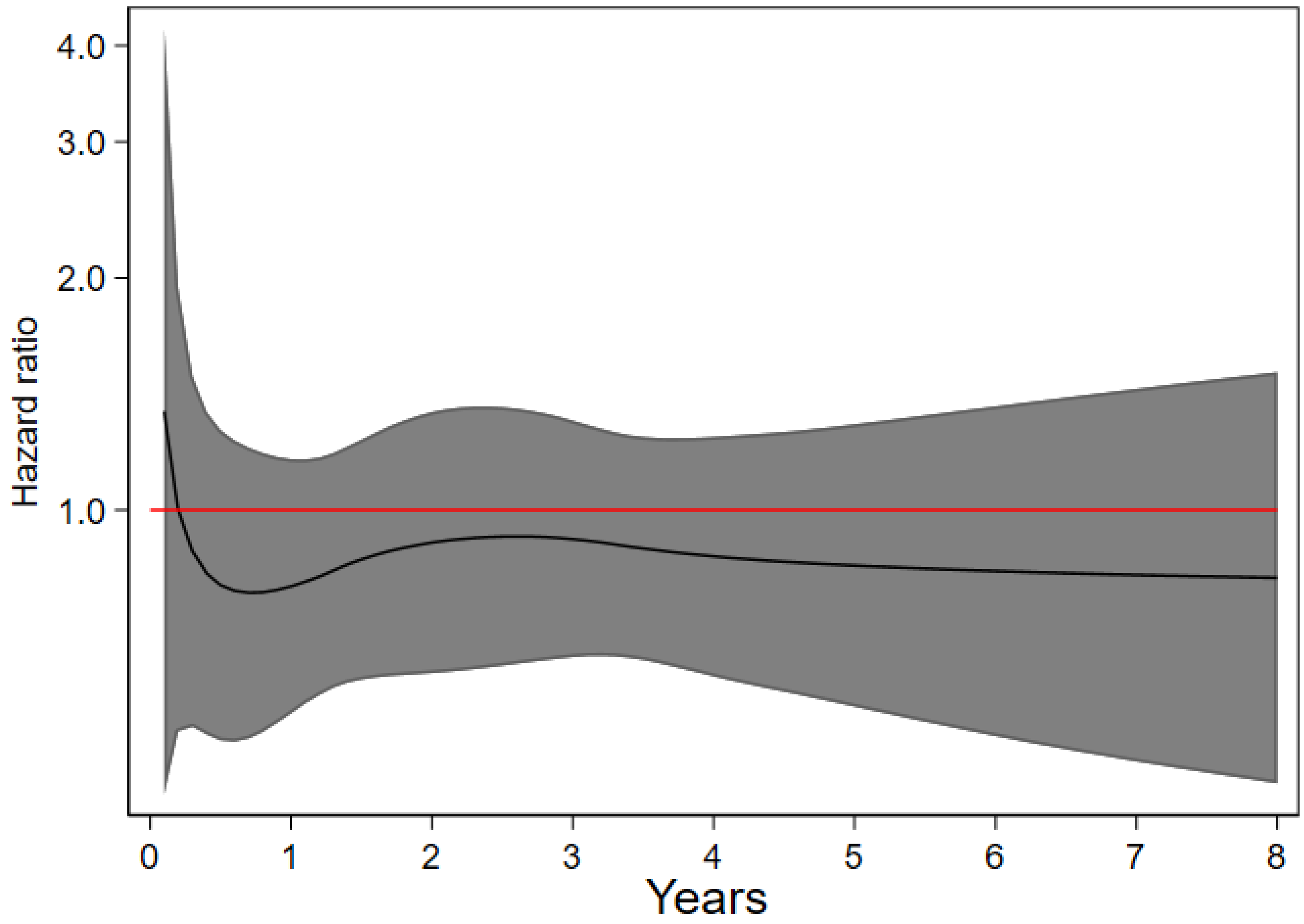

Supplementary Figure S13: Hazard ratios over time for the association between continued nitrogen-based bisphosphonate use and epithelial ovarian cancer diagnosis, compared to discontinued use, sensitivity analysis conditional on no diagnosis during the first year of follow-up (model S2.2).

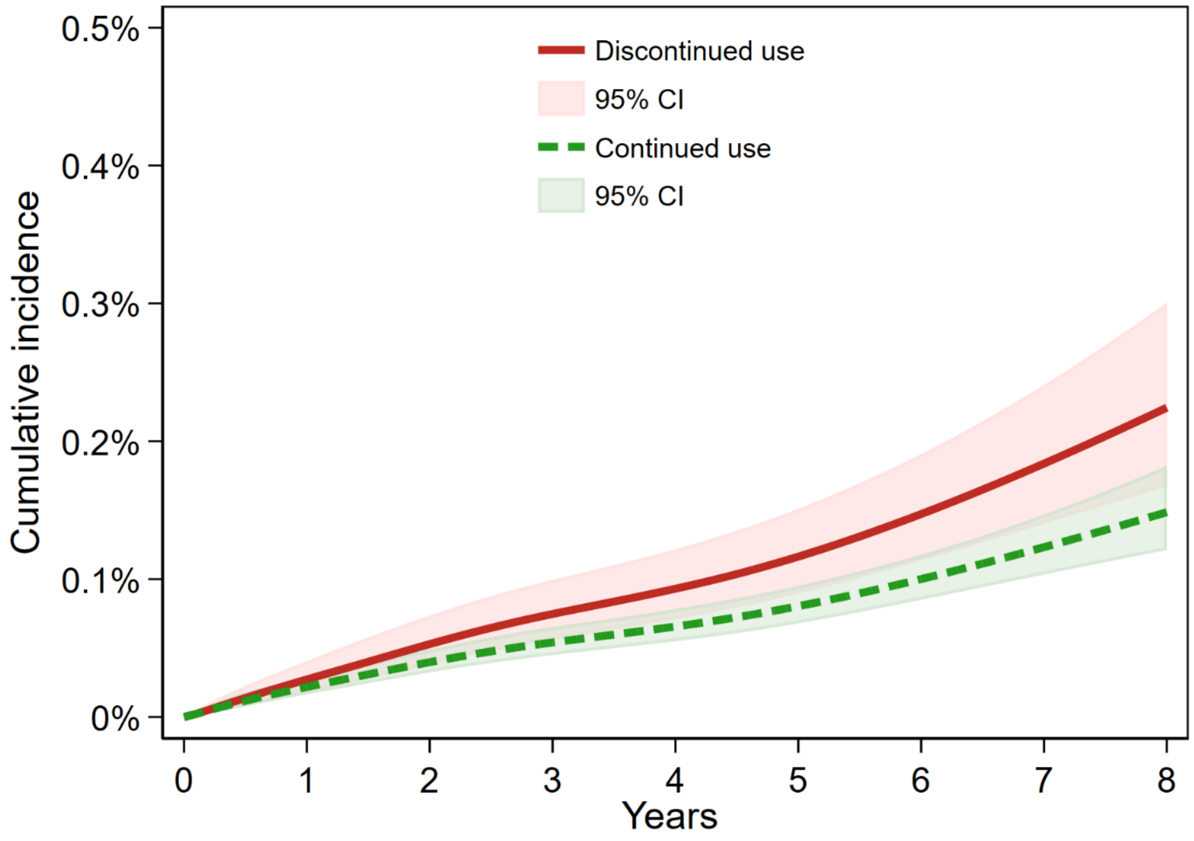

Number at risk

	0	1	2	3	4	5	6	7	8
Discontinued use	64,018	56,439	48,771	40,876	32,436	22,873	15,623	7,215	0
Continued use	215,119	187,659	160,803	134,855	110,106	82,640	56,434	26,715	0

Supplementary Figure S14: Inverse probability weighted survival model for outcome of serous ovarian cancer diagnosis for those women with discontinued use or continued use and estimated 95% confidence intervals, sensitivity analysis conditional on no diagnosis during the first year of follow-up (model S2.2).

CI: confidence interval

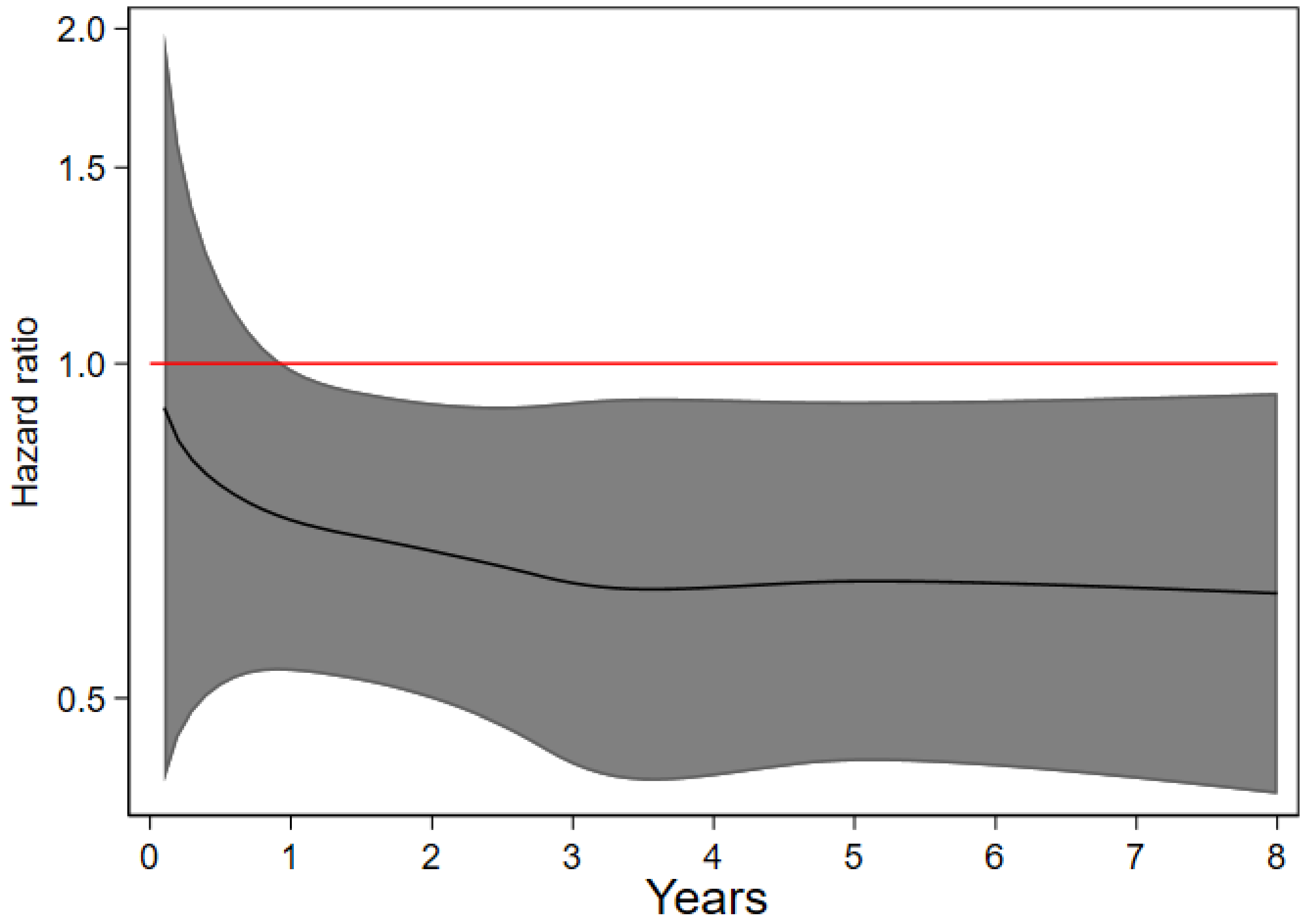

Supplementary Figure S15: Hazard ratios over time for the association between continued nitrogen-based bisphosphonate use and serous ovarian cancer diagnosis, compared to discontinued use, sensitivity analysis conditional on no diagnosis during the first year of follow-up (model S2.2).

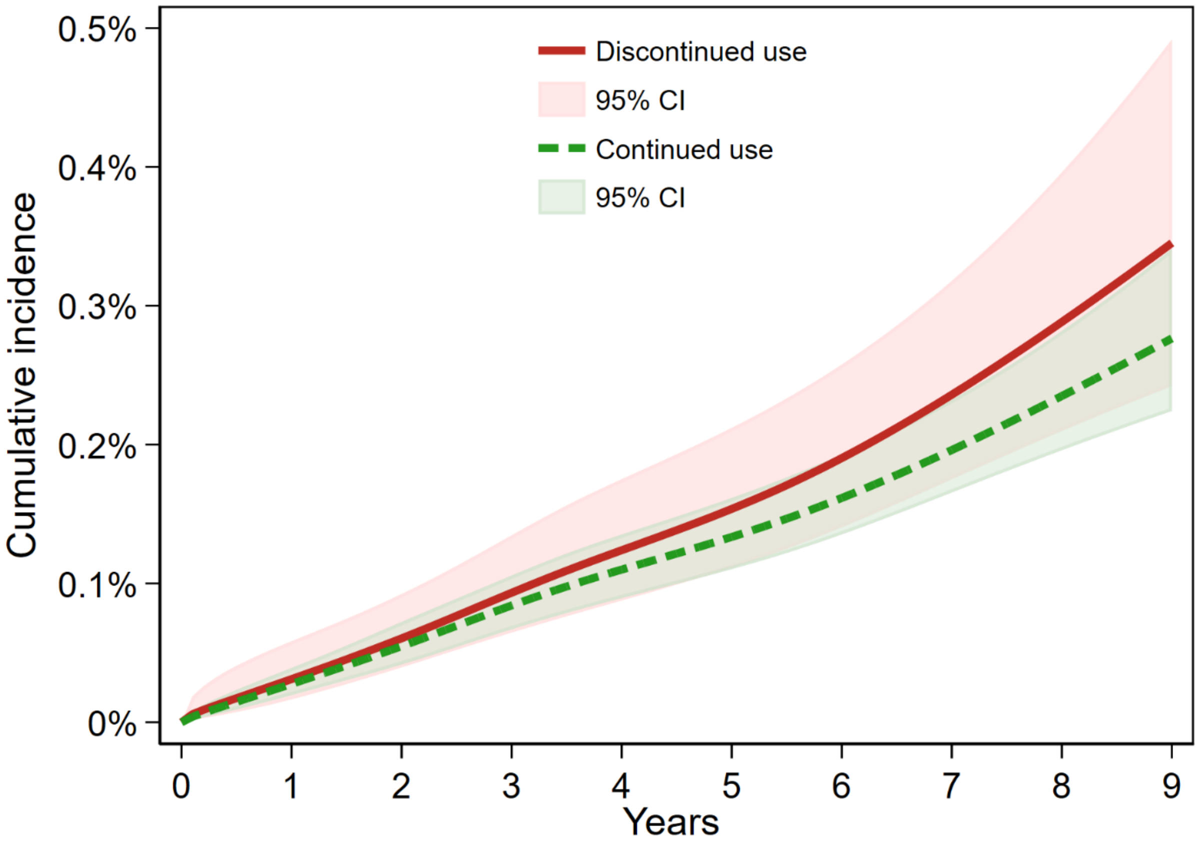

Number at risk

	0	1	2	3	4	5	6	7	8	9
Discontinued use	30,856	28,077	25,251	22,353	19,160	15,744	11,655	8,165	3,858	0
Continued use	97,762	88,619	78,899	69,166	59,466	50,530	40,224	28,008	13,447	0

Supplementary Figure S16: Inverse probability weighted survival model for outcome of epithelial ovarian cancer diagnosis for those women with discontinued use or continued use and estimated 95% confidence intervals, sensitivity analysis for women aged 70 and younger at first use (model S2.3).

CI: confidence interval

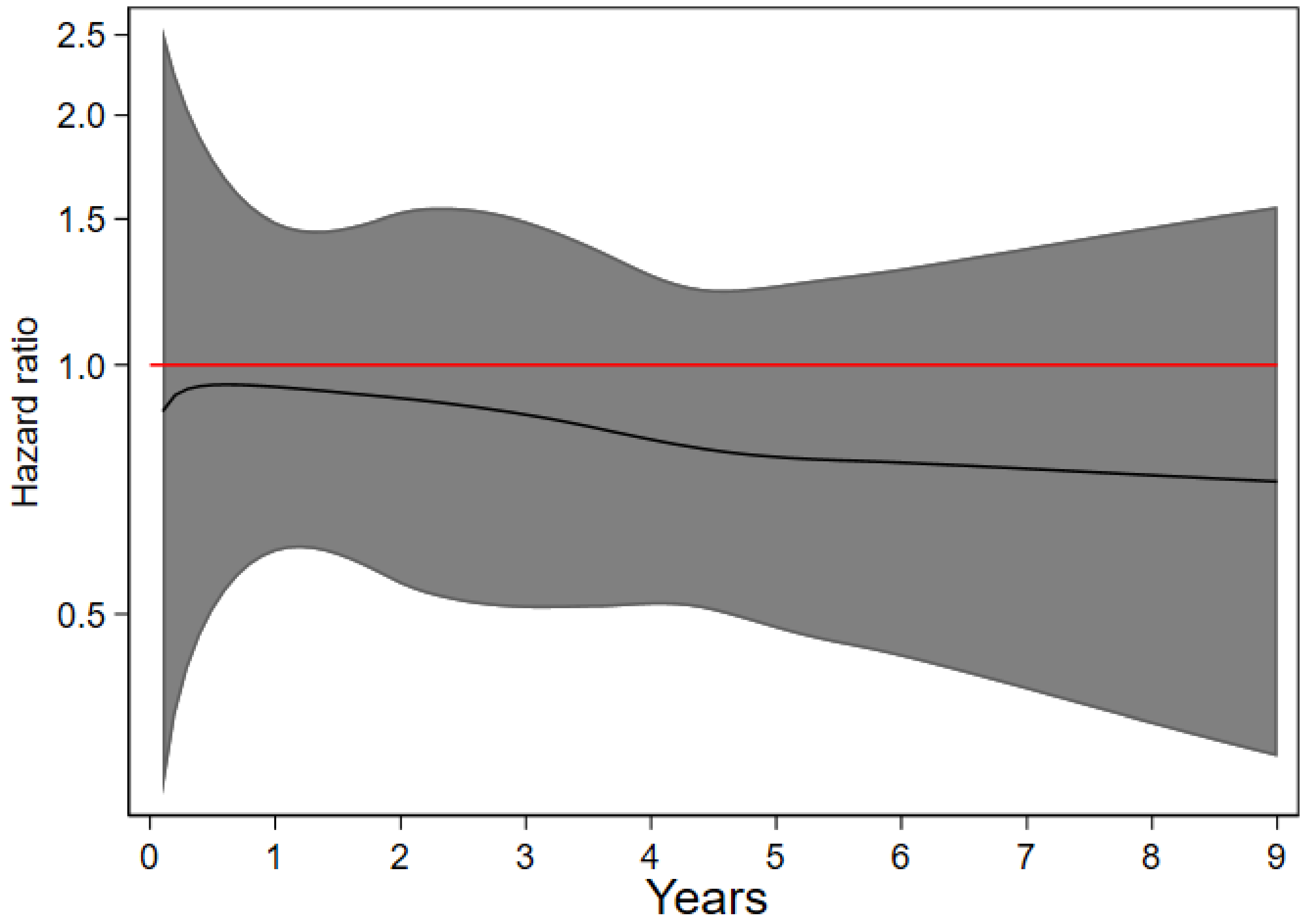

Supplementary Figure S17: Hazard ratios over time for the association between continued nitrogen-based bisphosphonate use and epithelial ovarian cancer diagnosis, compared to discontinued use, sensitivity analysis for women aged 70 and younger at first use (model S2.3).

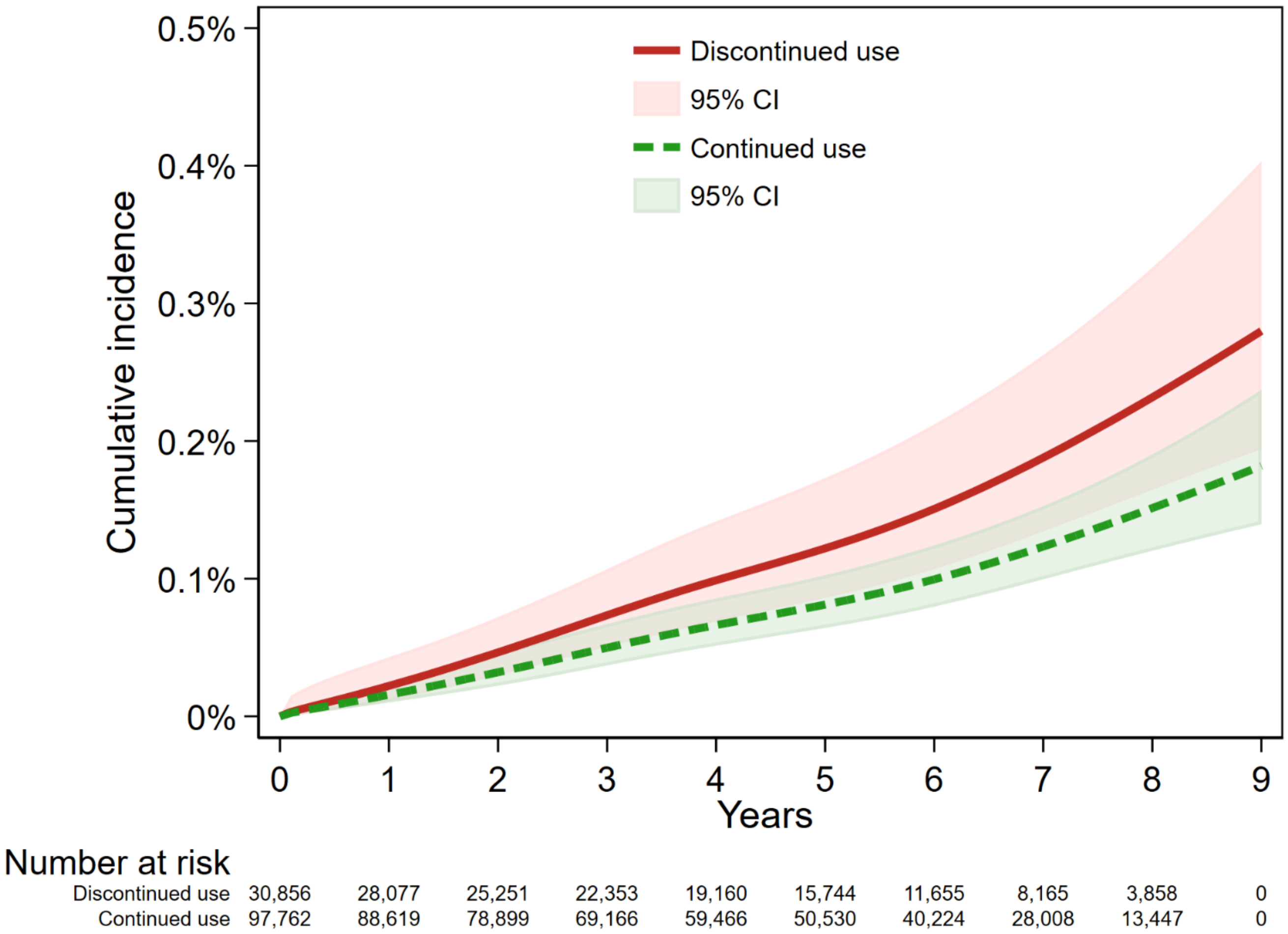

Supplementary Figure S18: Inverse probability weighted survival model for outcome of serous ovarian cancer diagnosis for those women with discontinued use or continued use and estimated 95% confidence intervals, sensitivity analysis for women aged 70 and younger at first use (model S2.3).

CI: confidence interval

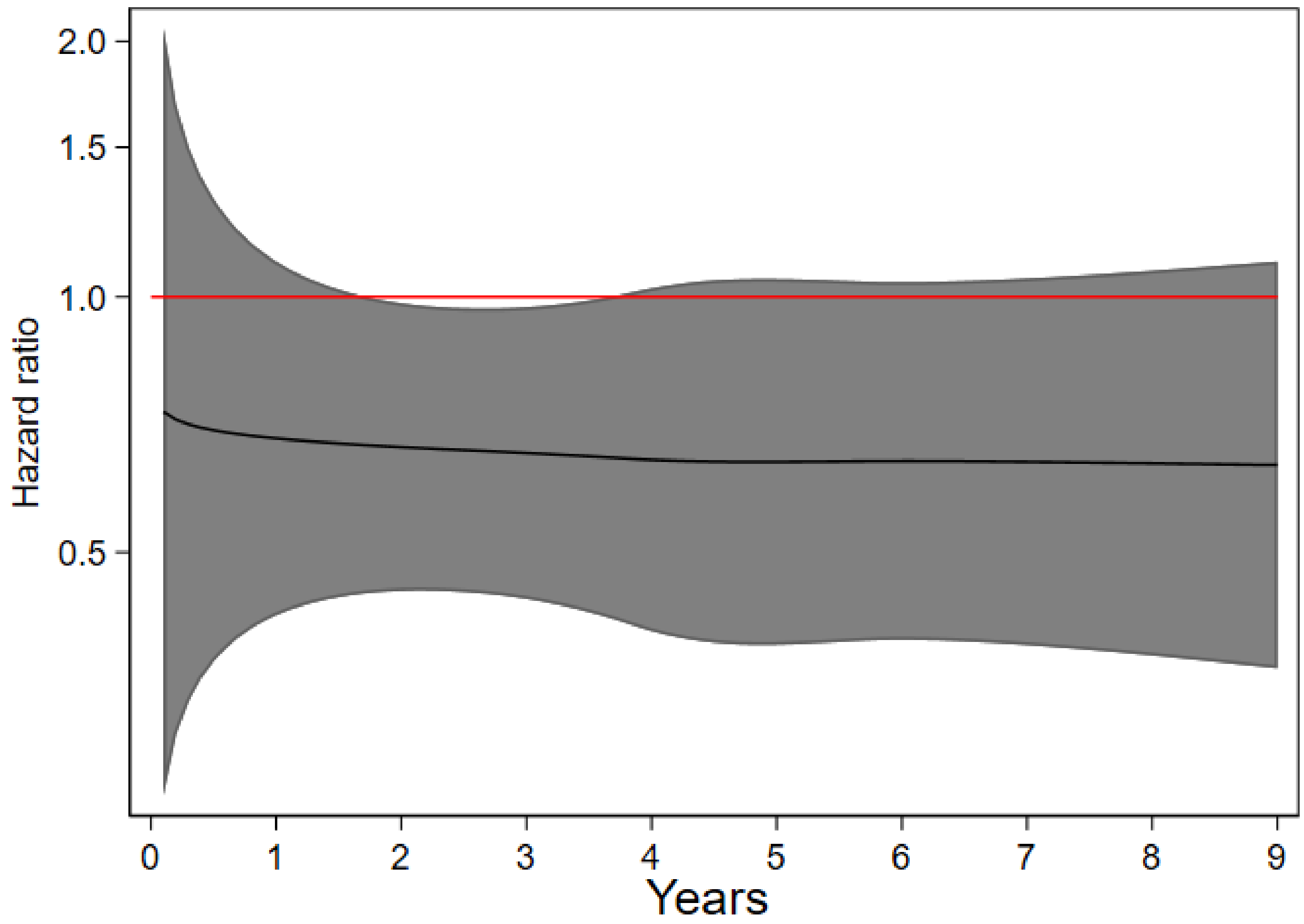

Supplementary Figure S19: Hazard ratios over time for the association between continued nitrogen-based bisphosphonate use and serous ovarian cancer diagnosis, compared to discontinued use, sensitivity analysis for women aged 70 and younger at first use (model S2.3).

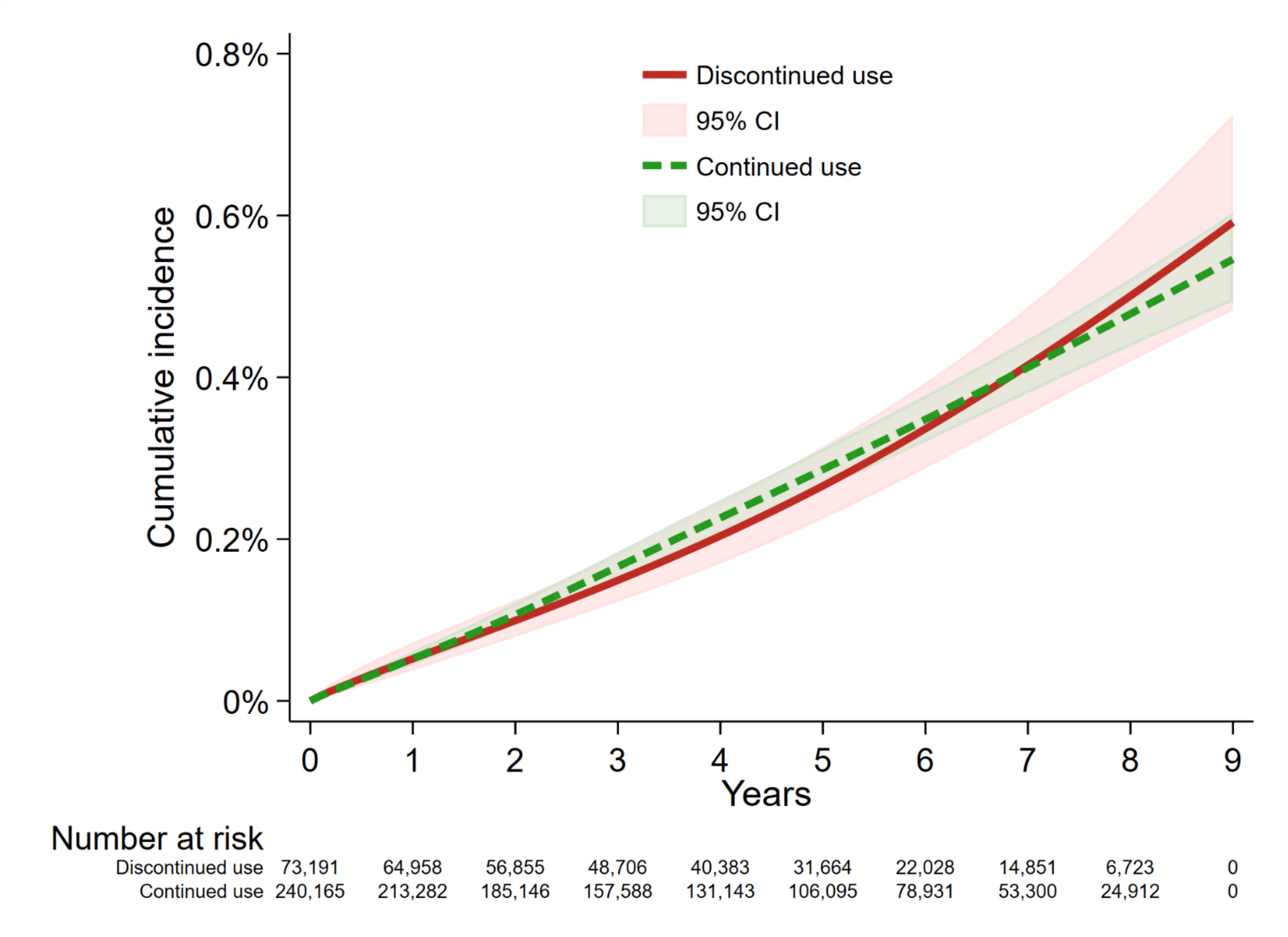

Supplementary Figure S20: Inverse probability weighted survival model for outcome of pancreatic cancer diagnosis for those women with discontinued use or continued use and estimated 95% confidence intervals (model S2.5).

CI: confidence interval

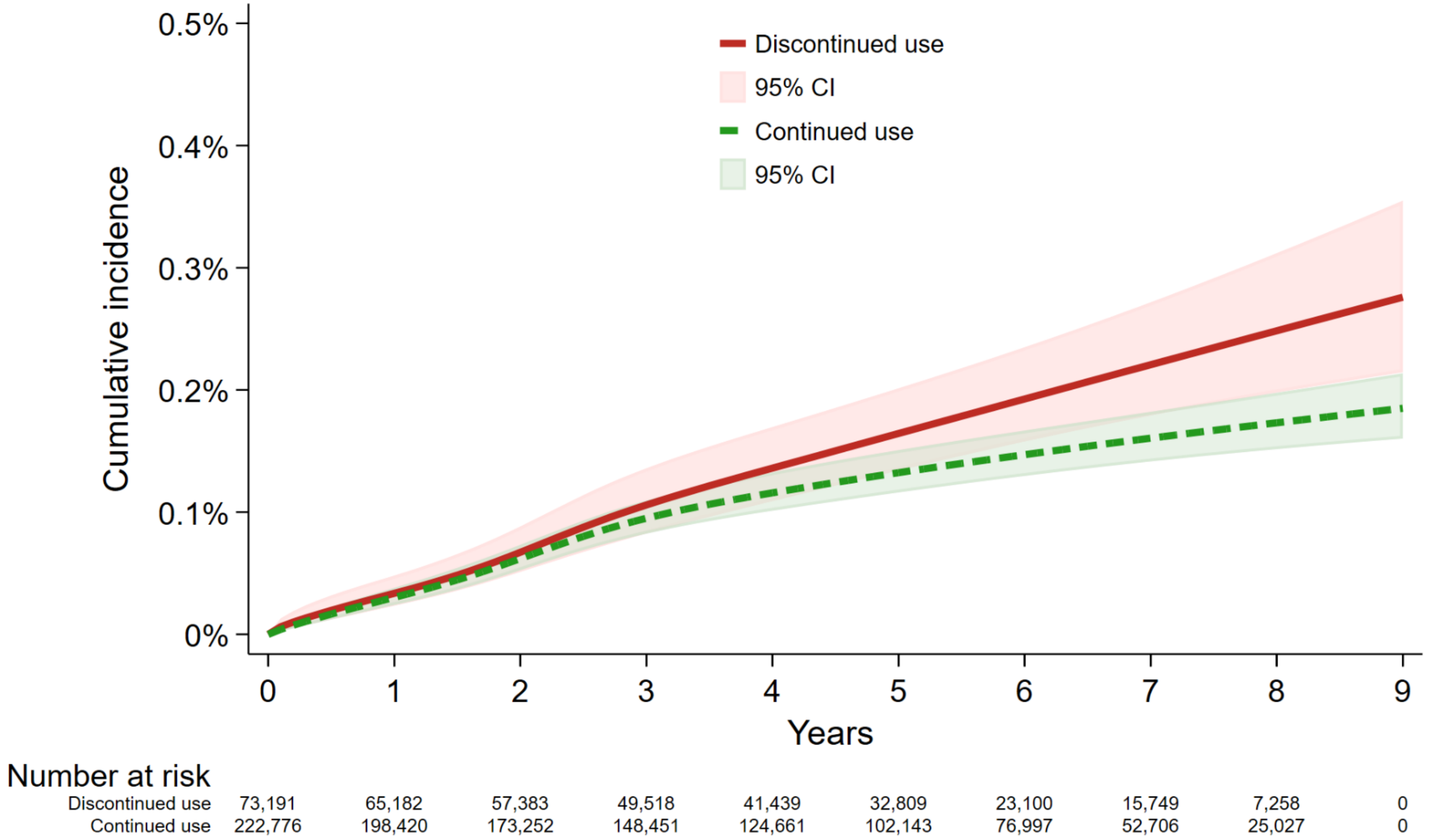

Supplementary Figure S21: Inverse probability weighted survival model for outcome of epithelial ovarian cancer diagnosis for those women with discontinued use or continued use and estimated 95% confidence intervals, sensitivity analysis for alternative treatment definition – per protocol analysis (model S2.6).

CI: confidence interval

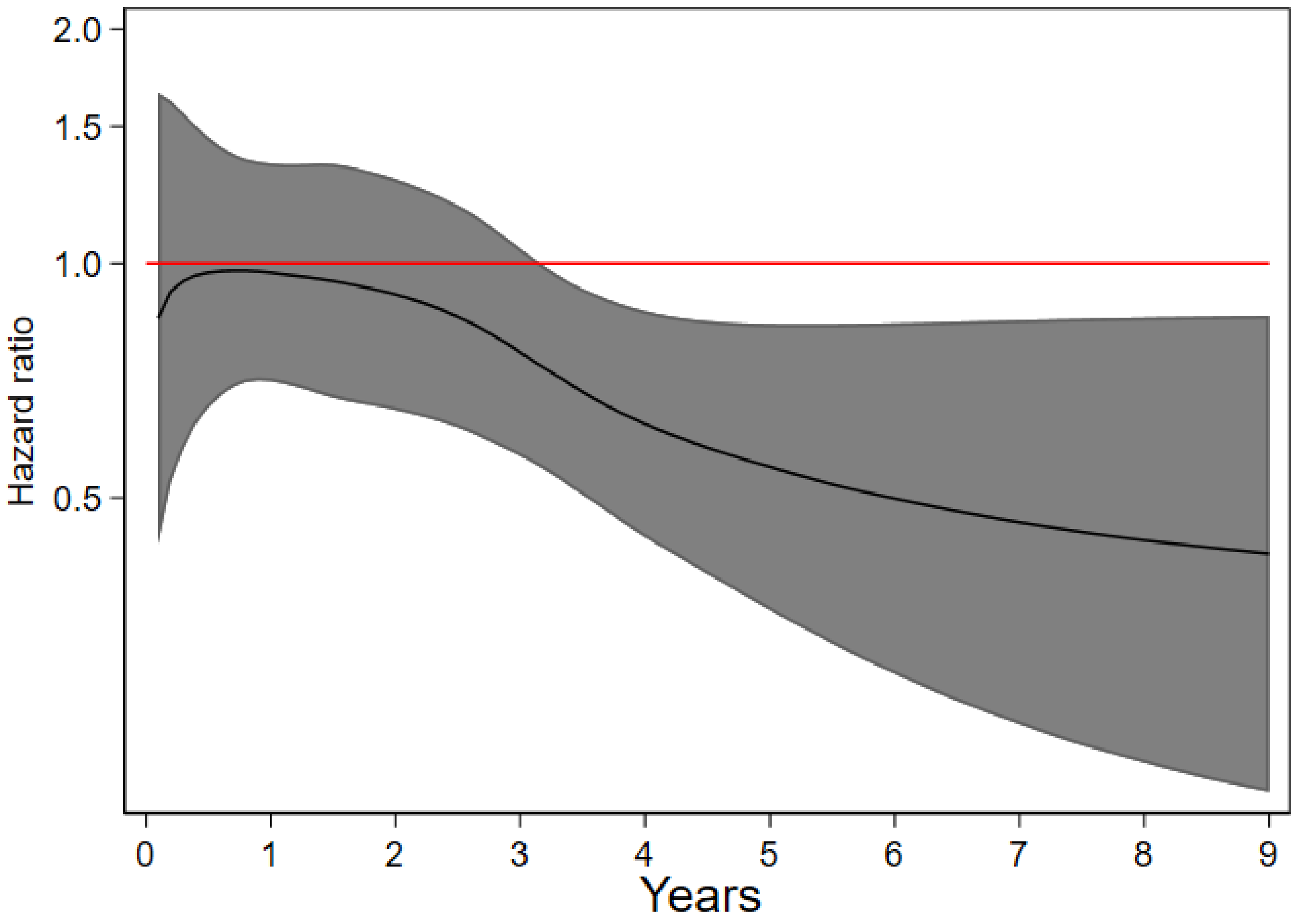

Supplementary Figure S22: Hazard ratios over time for the association between continued nitrogen-based bisphosphonate use and epithelial ovarian cancer diagnosis, compared to discontinued use, sensitivity analysis for alternative treatment definition – per protocol analysis (model S2.6).

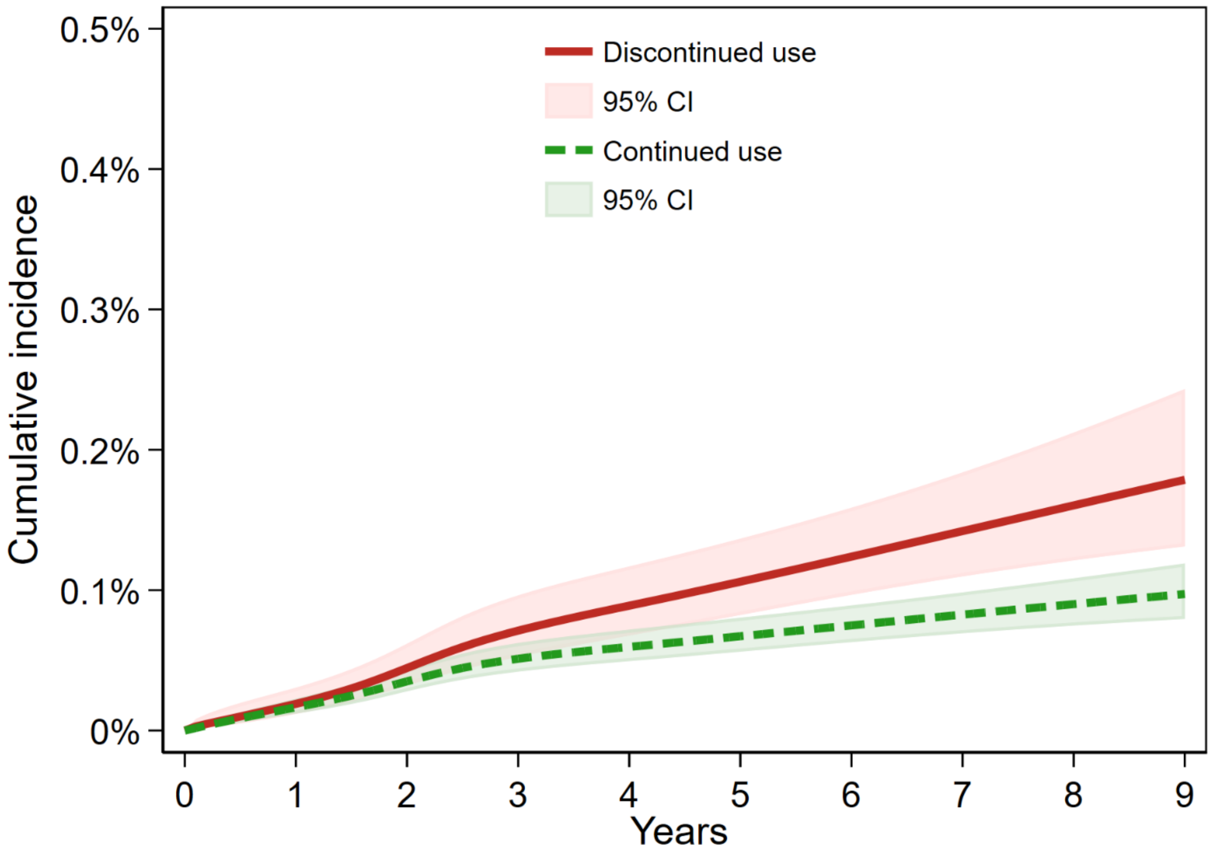

Number at risk

	0	1	2	3	4	5	6	7	8	9
Discontinued use	73,191	65,182	57,383	49,518	41,439	32,809	23,100	15,749	7,258	0
Continued use	222,776	198,420	173,252	148,451	124,661	102,143	76,997	52,706	25,027	0

Supplementary Figure S23: Inverse probability weighted survival model for outcome of serous ovarian cancer diagnosis for those women with discontinued use or continued use and estimated 95% confidence intervals, sensitivity analysis for alternative treatment definition – per protocol analysis (model S2.6).

CI: confidence interval

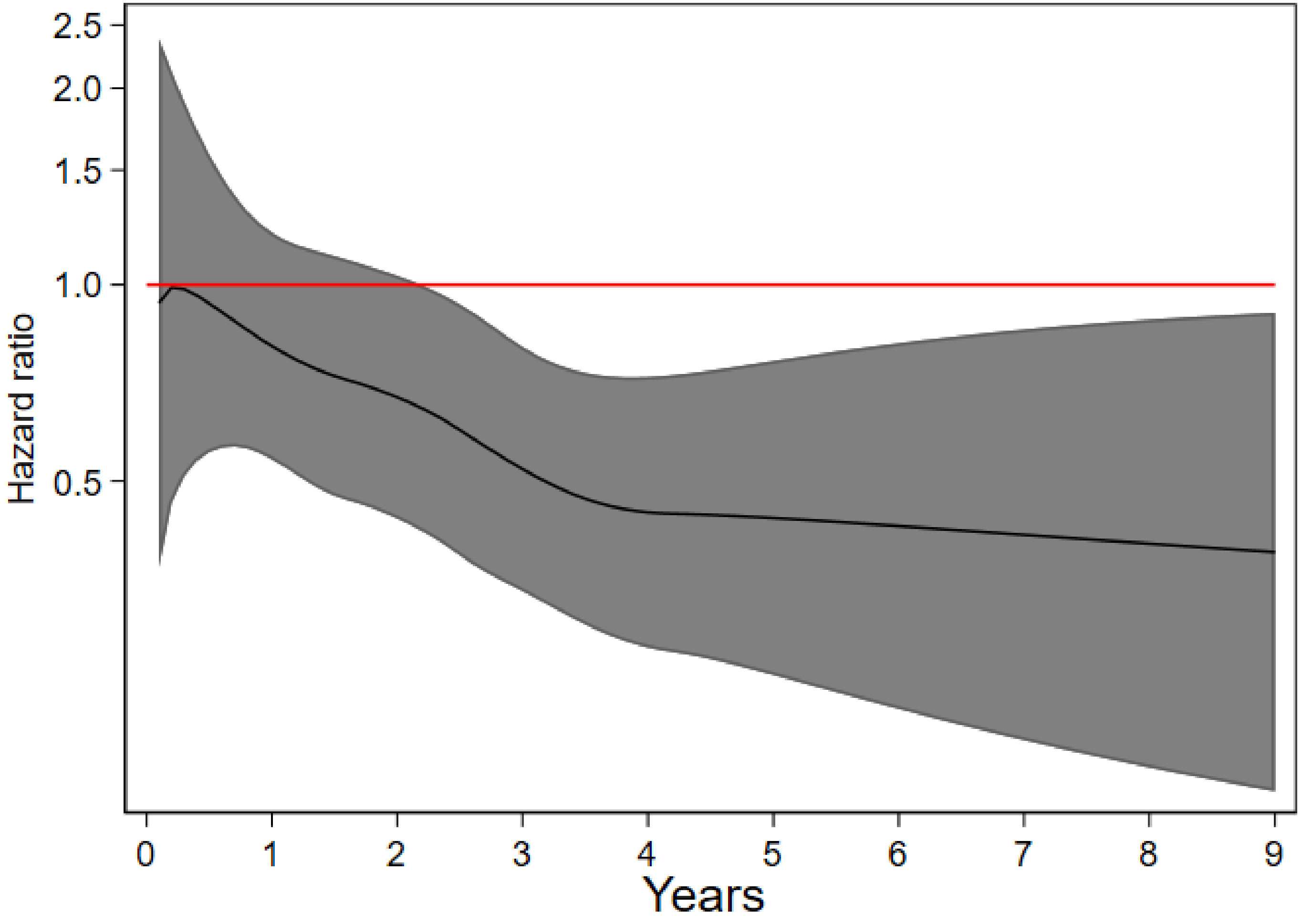

Supplementary Figure S24: Hazard ratios over time for the association between continued nitrogen-based bisphosphonate use and serous ovarian cancer diagnosis, compared to discontinued use, sensitivity analysis for alternative treatment definition (model S2.6).

References

1. SEIFA 2011: Australian Bureau of Statistics; 2011 [updated 19 September 2013. http://www.abs.gov.au/websitedbs/censushome.nsf/home/seifa2011?opendocument&navpos=260. (19 September 2019, date last accessed).

2. ABS Geography Publications: Australian Bureau of Statistics; 2017 [updated 21 April 2017. http://www.abs.gov.au/websitedbs/D3310114.nsf/home/ABS+Geography+Publications. (19 September 2019, date last accessed).

3. Australian Government Department of Health and Aged Care, The Pharmaceutical Benefits Scheme, Fees, Patient Contributions and Safety Net Thresholds 2022. https://www.pbs.gov.au/info/healthpro/explanatory-notes/front/fee. (11 November 2022, date last accessed).

4. Lu CY, Barratt J, Vitry A, Roughead E. Charlson and Rx-Risk comorbidity indices were predictive of mortality in the Australian health care setting. J Clin Epidemiol. 2011;64(2):223-8.

5. Pratt NL, Kerr M, Barratt JD, et al. The validity of the Rx-Risk Comorbidity Index using medicines mapped to the Anatomical Therapeutic Chemical (ATC) Classification System. BMJ Open. 2018;8(4):e021122.

6. Dodd S, Williamson P, White IR. Adjustment for treatment changes in epilepsy trials: A comparison of causal methods for time-to-event outcomes. Stat Methods Med Res. 2019;28(3):717-33.

7. Li YY, Gao LJ, Zhang YX, et al. Bisphosphonates and risk of cancers: a systematic review and meta-analysis. Br J Cancer. 2020;123(10):1570-81.

8. ATC/DDD Index: WHO Collaborating Centre for Drug Statistics Methodology, Norgewian Institute of Public Health; 2019 updated 13/12/2018. https://www.whocc.no/atc_ddd_index/. (2 April 2019, date last accessed).

9. Matz M, Coleman MP, Carreira H, et al. Worldwide comparison of ovarian cancer survival: Histological group and stage at diagnosis (CONCORD-2). Gynecol Oncol. 2017;144(2):396-404.
